# Stable isotopes in global lakes integrate catchment and climatic controls on evaporation

**DOI:** 10.1038/s41467-021-27569-x

**Published:** 2021-12-10

**Authors:** Yuliya Vystavna, Astrid Harjung, Lucilena R. Monteiro, Ioannis Matiatos, Leonard I. Wassenaar

**Affiliations:** International Atomic Energy Agency, Isotope Hydrology Section, Vienna International Centre, PO Box 100, 1400 Vienna, Austria

**Keywords:** Environmental sciences, Hydrology, Limnology

## Abstract

Global warming is considered a major threat to Earth’s lakes water budgets and quality. However, flow regulation, over-exploitation, lack of hydrological data, and disparate evaluation methods hamper comparative global estimates of lake vulnerability to evaporation. We have analyzed the stable isotope composition of 1257 global lakes and we find that most lakes depend on precipitation and groundwater recharge subsequently altered by catchment and lake evaporation processes. Isotope mass-balance modeling shows that ca. 20% of water inflow in global lakes is lost through evaporation and ca. 10% of lakes in arid and temperate zones experience extreme evaporative losses >40 % of the total inflow. Precipitation amount, limnicity, wind speed, relative humidity, and solar radiation are predominant controls on lake isotope composition and evaporation, regardless of the climatic zone. The promotion of systematic global isotopic monitoring of Earth’s lakes provides a direct and comparative approach to detect the impacts of climatic and catchment-scale changes on water-balance and evaporation trends.

## Abstract (web summary)

An isotope synthesis of 1257 global lakes revealed on average 20% of inflow is lost to evaporation, but 10% of Earth’s lakes show extreme evaporative losses. Stable water isotope monitoring is an effective way to detect comparative climatic and catchment-scale impacts on lake water-balance budgets.

## Introduction

Lakes comprise ca. 87% of available global surficial freshwater storage and are sentinels of climatically driven catchment and surface water evaporation impacts^1,2^. Lake evaporation is driven by a suite of hydroclimatic and environmental factors including solar radiation, lake and catchment surface area, climate type, albedo, wind speed, relative humidity, air temperature, and heat storage^1–4^. Evaporative loss potential from lakes is often predicted using multi-parametric meteorological models (Penman−Monteith, Priestley−Taylor Equations), energy-budgets (Bowen ratio), lake simulators, or lake evaporation pans^5–7^. Stable isotopes are used to estimate evaporative loss by measuring the ^18^O/^16^O and ^2^H/H ratios of the lake water compared to in- and outflows (e.g., precipitation, atmospheric vapor, rivers, and groundwater) and using isotope mass-balance models to quantify evaporation to inflow (E/I) ratios^8–13^. Lakes undergoing evaporation exhibit distinctive trends in their ^18^O/^16^O vs ^2^H/H ratios away from the precipitation input, and the characteristic “local evaporation lines” (LEL) are assessed by using air temperature and relative humidity via the Craig−Gordon evaporation model^8,10,11,14,15^. For meteorological-based evaporation prediction models, crucial information obtainable from stable isotopes to help constrain variables driving lake water evaporation are rarely used, and conversely, isotope mass-balance models do not incorporate a wide range of hydroclimatic or environmental variables. In both approaches, a full suite of variables of lake evaporation within the lake-catchment system framework are generally overlooked (e.g., limnicity, solar radiation, wind speed) or are qualitatively acknowledged^13^. Recent efforts to explain global variations in the *δ*^18^O of lakes focused on catchment-scale evapotranspiration patterns^15^, however, no comparative causal assessment using multi-component variables that control the stable isotope composition *(δ*^18^O_L_ and *δ*^2^H_L_) of global lakes has been undertaken to date. One advantage of using stable isotopes is they provide a globally comparative metric (as opposed to often incomparable international hydrometric data) to quantify lake evaporation, not only from water surfaces, but also to disentangle the relevance and commonalities of hydroclimatic variables within lake-catchment systems^8,10,11,14,15^.

Our hypothesis was that the stable isotope compositions of Earth’s lakes are controlled by precipitation amount-weighted inputs and evaporation losses of the lake inflows, which are controlled by distinctive combinations of regional climatic and catchment-scale environmental factors. We anticipated that lake isotope data represents the net evaporative enrichment, and would reveal common, quantifiable, and comparative evaporation losses for global lakes relative to inflows. To test our hypothesis, we assembled a curated dataset of 7415 stable isotope measurements from 1257 large-to-small lakes across Earth’s continents spanning diverse geographical and climatic zones: tropical, arid, temperate, continental, and polar^16,17^. Details about lake selection criteria and data curation are presented in “Methods” section. Each lake’s isotopic composition was normalized to its catchment-weighted precipitation input^12^, evaluated, and modeled for E/I by using an array of potential drivers of lake-catchment evaporation (air temperature, relative humidity, solar radiation, etc.) obtained from global geospatial datasets.

## Results and discussion

### Variation of lake isotopic composition with climate

**Fig. 1:**
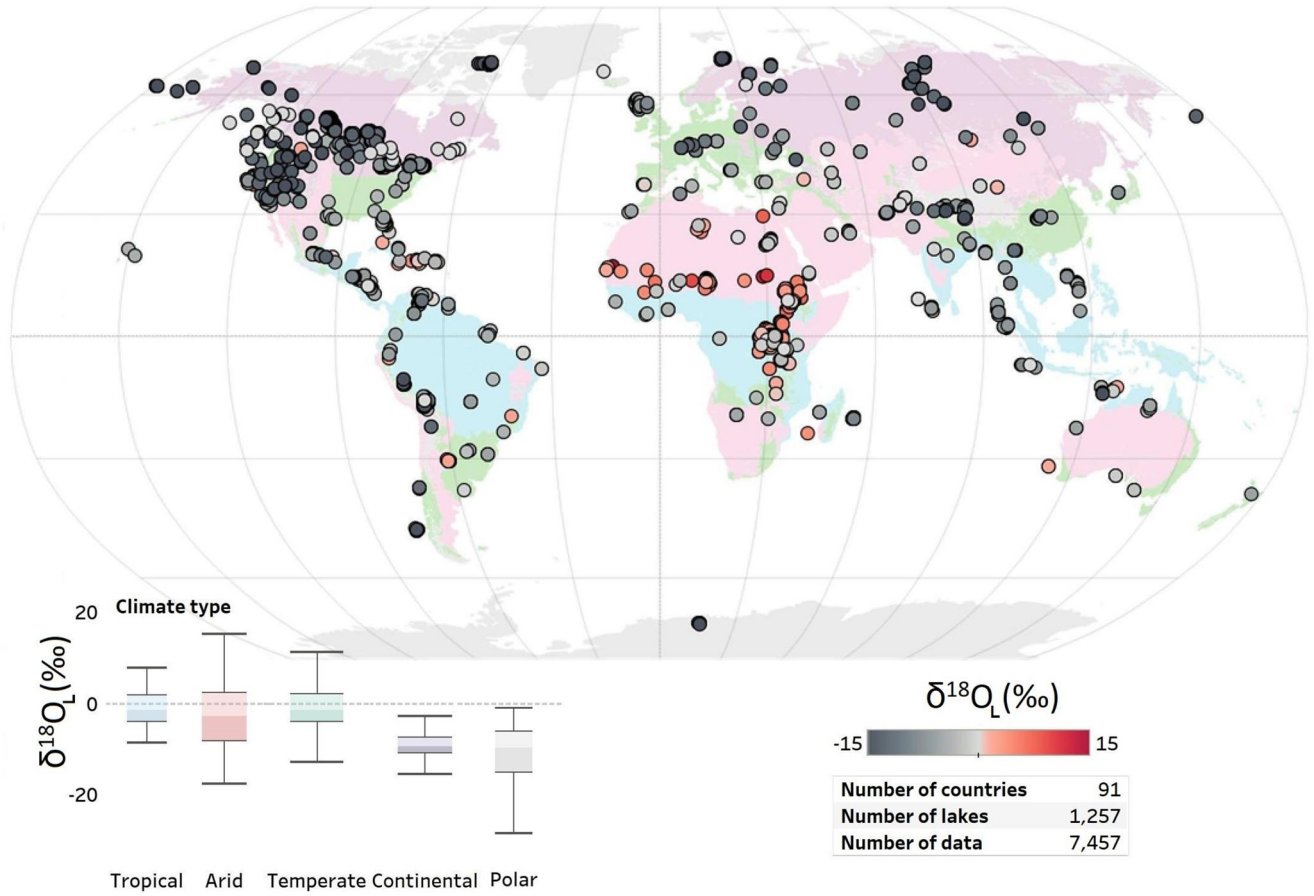
Oxygen isotopic composition of global lakes. Distribution of *δ*^18^O_L_ composition (*n* = 7457 data points) in 1257 lakes around the globe and in climatic zones based on the Köppen−Geiger climate classification^16, 17^. Median and ranges of *δ*^18^O_L_ for all lakes by climatic zone are depicted in box-and-whiskers plots. The climate map was generated according to Kottek et al.^17^.

Several broad geospatial isotope patterns for Earth’s lakes were evident (Fig. 1). Tropical lakes generally had more positive median *δ*^18^O_L_ values (−1.3‰) compared to high latitude polar lakes (−9.8‰) (Fig. 1 and Fig. S1 for *δ*^2^H in the Supplementary Information) and all lakes broadly mirrored the expected relationship between *δ*^18^Ο_L_ and absolute latitude, but with more positive *δ* values than precipitation (*δ*^18^O_L_ is −6.9 ‰ ± 6.2, *n* = 1238 in lakes and *δ*^18^O_P_ is −12.3‰ ± 4.8, *n* = 1257 in precipitation, *p*-value < 0.001, t-test) (Figs. S2 and S3 in the Supplementary information).

Lake water undergoing evaporation becomes enriched in the heavy isotopes along the LEL with lower slopes (*m* = 4–6) that diverge from the Global Meteoric Water Line (GMWL, *m* = 8)^11,18^. The intersection of each LEL with the GMWL provides an estimate of the lake water origin (input water), whereas the displacement distance from the input value along the LEL reveals the extent of evaporation loss of the lake in relation to the inflow, as expressed by the E/I ratio^15,19^.

**Fig. 2:**
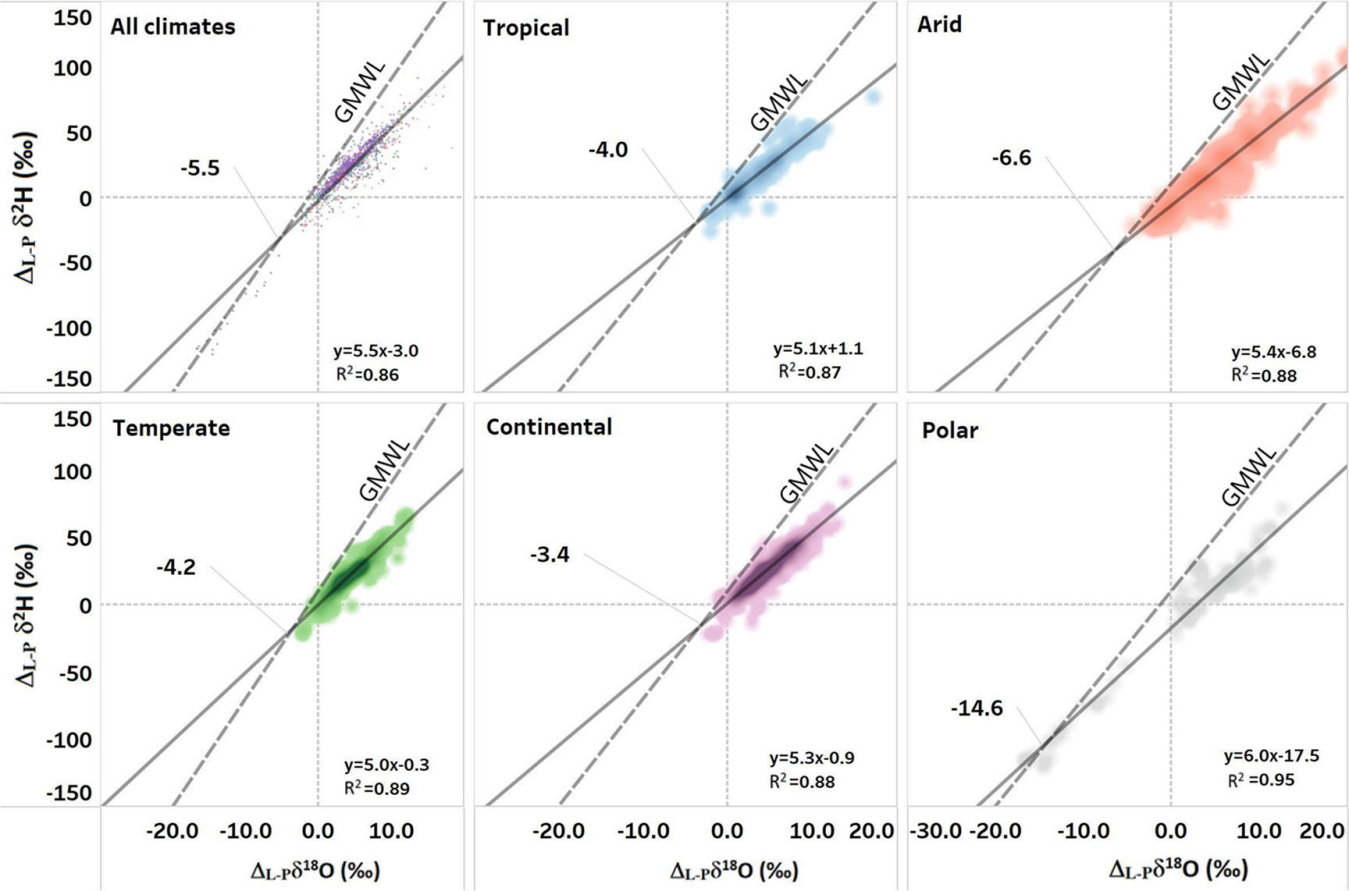
Isotope evaporation lines for global lakes. The local evaporation lines (LEL) and their coefficients of determination (*R*^2^) based on the isotopic values of lakes normalized to their weighted catchment precipitation input (Δ_L-P_δ^18^O and Δ_L-P_δ^2^H) and the intersection of these lines with the global meteoric water line (GMWL). Intersection values refer to *δ*^18^O values.

At the global scale, the lake water LEL intersection for Δ_L-P_*δ*^18^O was slightly lower (−5.5‰) than oceanic derived precipitation, which ranges from 0 to −5‰ (Fig. 2)^18^. The higher Δ_L-P_*δ*^18^O values (−3.4, −4.0, and −4.2‰) of the LEL intersection for continental, temperate, and tropical climate lakes are indicative of significant water inputs from isotopically enriched water storage in the lake catchments. These intersection isotopic values were close to that of local groundwater and to sea water in tropical and temperate climate (from 0 to −6‰)^20,21^. The lower intersection of Δ_L-P_*δ*^18^O values (−6.6‰) in arid zone lakes suggests a greater dependency on groundwater storage with isotopic compositions ranging from −6 to −9‰, suggesting recharge from higher elevation or older groundwater recharged under cooler climate conditions, or from cold season recharge bias (Fig. 2)^21^. The higher LEL intersection for continental climate lakes indicates that water originates from shallow water storage (wetlands, soil water), which are highly diverse in continental zones and where wetlands are well connected to river and lakes systems^22–24^. The low LEL intersection (−14.6‰) for polar lakes is indicative for water inputs from glacier, ice, snowmelt, or that represents paleoclimate conditions from permafrost melt (Fig. 2)^25,26^.

The LEL slopes varied from 5.0 in temperate zones to 6.0 for polar lakes (Fig. 2). Altogether, the global lake-catchment water Δ_L-P_*δ*^2^H versus Δ_L-P_*δ*^18^O line had a slope of 5.5 (Fig. 2). Variance in the Δ_L-P_*δ*^2^H versus Δ_L-P_*δ*^18^O relationships stems from isotope fractionation during phase changes, mainly from evaporation and mixing of waters of different origin^10^. The slopes of these global lake-catchment evaporation lines differed significantly from localized lake-modeled slopes presented in some other studies^10,11,27^. The Craig−Gordon model for evaporation shows that the slopes of the LEL are predictable by relative humidity and the isotopic composition of the vapor layer, which is temperature-dependent and expressed by isotopic fractionation under equilibrium conditions. Using the Craig−Gordon model, Gibson et al. (2016) predicted slopes of <4.0 for low latitude, arid, and humid climate zone lakes based on temperature and humidity variables alone, whereas our global dataset revealed slopes for lakes in tropical, arid, and continental climates that were significantly higher than the theoretical predictions^28,29^. Generally, it is considered that the mean isotopic composition of the atmospheric vapor is not linked to that of precipitation, especially in arid areas, where precipitation is rare, relative humidity is low and thus in the end the influence of the atmospheric vapor is limited and results in heavy isotope enrichment and greater range of the isotopic variation^11^. However, arid climates include a range of sub-climate categories, i.e., hot, and cold desert^16^, indicating that additional climate parameters inside of the individual sub-category can significantly impact evaporation processes. For example, the presence of the vapor layer can be eliminated at high wind speeds, and thus a kinetic evaporation process will prevail over the equilibrium one, yielding slightly higher LEL slopes^8,30^. The higher slopes observed in polar lakes versus Craig−Gordon model predicted values can be attributed to the influence of additional parameters affecting the evaporation process (e.g., albedo) that are not taken into consideration in isotope mass-balance models.

**Fig. 3:**
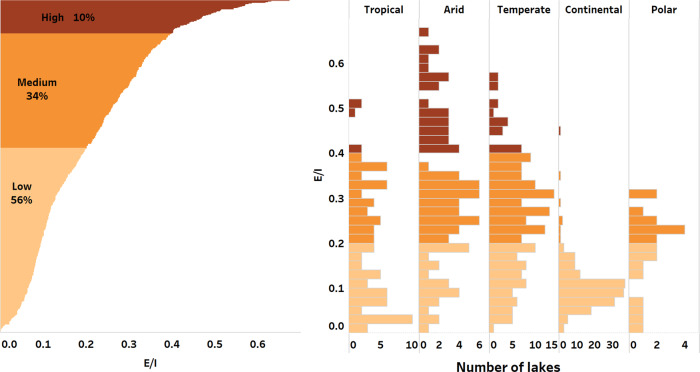
Isotope-based evaporation losses from global lakes. Relative proportion of Earth’s lakes evaporation losses (E/I) and by climatic zone.

Globally, based on the E/I values of lakes with sufficient data, around 10% of Earth’s lakes had high evaporation losses (E/I > 40%), but the majority (56%) of lakes showed lower evaporation losses (E/I < 20%) (Fig. 3).

Lakes in arid and temperate climate zones had highest evaporation losses (ca. 50% of lakes in these climatic groups) (Fig. 3). Overall, lakes with highest evaporation losses were characterized by having the highest median catchment (1083 km^2^ versus 61 km^2^) and lake surface areas (24 km^2^ versus 0.4 km^2^) and the highest evaporative isotopic enrichment *δ* values in relation to lakes with the lowest E/I (Fig. S4 and Table S1 in the Supplementary Information). The mean E/I value for Earth’s global lakes was 0.2, indicating that at the global scale, around 20% of water inputs into the lakes are lost by evaporation.

### Climate and catchment-scale processes effect lake evaporation status

**Fig. 4:**
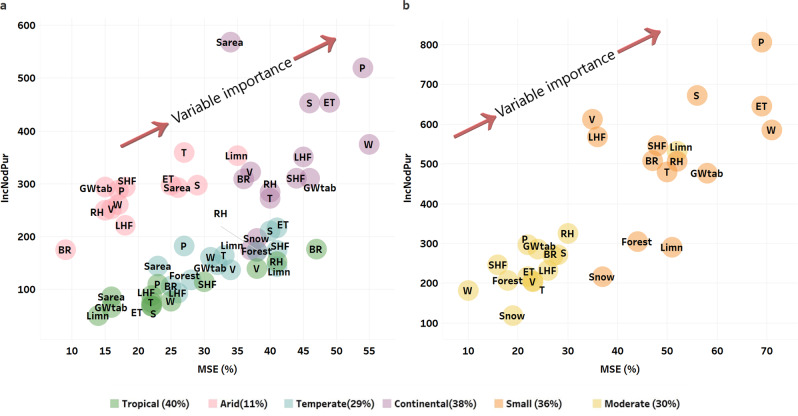
Determinants of evaporative isotopic enrichment of global lakes. Random forest model results for variables that control the evaporative enrichment in lakes (Δ_L-P_*δ*^18^O) with respect to climatic zone (**a**) and lake size (**b**). Model accuracy was tested using the Mean Decrease Accuracy (MSE, %) and the Mean Decrease Gini (IncNodPur, *y*-axis). Environmental variables used in the random forest model: Bowen ratio (BR, dimensionless), evapotranspiration (ET, mm), forest coverage in the catchment (Forest, %), groundwater table (GWtab, cm), catchment limnicity (Limn, %), latent heat fluxes (LHF, W/m^2^), precipitation amount (P, mm), relative humidity (RH, %), lake surface area (Sarea, km^2^), sensitive heat fluxes (SHF, W/m^2^), snow coverage in the catchment (Snow, %), solar radiation (S, kJ/m^2^), air temperature (*T*, °C), vapor saturation (V, kPa) and wind speed (W, m/s). Model performance by zone and lake size (*R*^2^) is presented as % in the legend. Variable importance is indicated by the arrow direction.

Overall, the differences among LEL slopes and E/I for lakes among climate types result from the influence of a wide range of regional-scale climate conditions and their concurrent influence on the evaporation processes occurring in the catchments. Lake catchments at lower latitudes absorb higher amounts of solar energy^2^ and are influenced by greater moisture availability from tropical cyclones and moisture feedback from lakes into the atmosphere, which strongly affect the lake isotopic composition^28,31^. This was affirmed by results of the Random Forest (RF) model for tropical lakes, where relative humidity and Bowen ratio variables explained the evaporative enrichment of these lower latitude lakes (Δ_L-P_*δ*^18^O and Δ_L-P_*δ*^2^H) (Fig. 4 for Δ_L-P_*δ*^18^O and Fig. S5 in the Supplementary Information for Δ_L-P_*δ*^2^H).

Solar radiation was a top climatic explanatory variable for Δ_L-P_*δ*^18^O for arid zone lakes, whereas evapotranspiration best explained evaporative enrichment in temperate climate zone lakes. Wind speed was a strong explanatory variable of the evaporative isotopic enrichment for continental lakes (Fig. 4). Selected catchment and climate variables in the (few) polar lakes had generally low explanatory power for Δ_L-P_*δ*^18^O (*R*^2^ = 4 %) since the isotopic composition of these lakes is mainly indicative of water inputs than evaporation. Lake recharge in polar regions originates largely from the melting of the cryosphere (snow, glacier, ice, soil permafrost)^25^, and polar lakes have high albedo from snow and ice cover, which reduces the absorption of solar energy and the exchange of water between the watershed and atmosphere^2^.

Except for lakes in arid climate zones, air temperature was not a dominant climatic variable that controlled lake evaporation (Fig. 4). This agreed with recent studies^1,2^ that highlighted a stronger control by heat transfer parameters (solar radiation and Bowen ratio) on lake evaporation, rather than air temperature. Catchment-scale parameters were found also to be important as explanatory variables for the isotopic composition of lakes in arid (limnicity and surface area) and temperate (precipitation amount and groundwater table) zones (Fig. 4).

Considering lakes by their surface area, we found evaporative enrichment in small lakes (<10 km^2^ of surface area) was best explained by catchment precipitation amount, evapotranspiration, and average wind speed. These findings can be elucidated by shorter lake water residence times and faster hydrological cycles mainly controlled by precipitation inputs and catchment-scale evapotranspiration^28^. The Δ_L-P_*δ*^18^O in moderate-sized lakes (from 100 to 500 km^2^) was best explained by limnicity, relative humidity, solar radiation, and Bowen ratio. The Δ_L-P_*δ*^18^O in great lakes (>500 km^2^ surface area) was poorly explained by the considered environmental parameters (*R*^2^ = 9%), suggesting that, owing to the long water residence times, the isotopic composition of large lakes cannot be easily explained or predicted by shorter-term climatic conditions. Additional factors, or a combination of parameters that impact the isotope signature for large lake-catchments (e.g., long-term land use changes, residence time, etc.), might improve the RF model performance, but cannot be easily accounted for on a large scale.

### Global lake-catchment evaporation drivers

Lake water sustained from various water-storage reservoirs in the catchment experience evaporation processes along the flow paths to the lake (from soil water, river, and surface water) and from the lake surface itself. At the global scale, evaporation losses accounted for ca. 20% of the water inflows into the lake and were found to be driven by a complex range of climate and catchments variables, whose interrelationships integrated the isotope values in the lakes and their distinctive LEL patterns. Among the many variables we considered, catchment (limnicity and precipitation amount), together with the key physical driving forces of water evaporation (air temperature, relative humidity, wind speed, and solar radiation) were the main variables that explained the isotopic composition of lakes with respect to climate type. Our findings also agreed with recent energy balance studies in global lakes^1,2^ where solar radiation is an important atmospheric driver of lake evaporation. However, our results showed that not only are climatic variables important, but a combination of climatic and landscape variables by climate zone and lake size also need to be considered to fully understand and predict lake evaporation processes. The energy available for lake water evaporation is modulated by surface water temperature and only partly driven by air temperature through sensible heat flux, incoming shortwave and longwave radiation, the proportion of solar irradiance absorbed at the lake surface (albedo), the advective sources of heat within the lake (for example snowmelt and groundwater), and changes in lake heat storage (such as through changes in lake stratification and mixing)^1,2^.

We showed that not only are relative humidity and air temperature indicative for predicting the isotopic composition of global lakes, but also variables related to these surface energy processes, that are incorporated in conventional evaporation models (e.g., Penman)^8,11,27^. Additionally, traditional isotope-enabled water balance studies generally focus on the lake itself, and rarely consider lake evaporation as an integrated part of the lake-catchment system. While predicted global warming may differentially impact the lake-watershed by involving higher evaporative losses from the lake surface, warming may also affect catchment-scale processes affecting the isotope composition of the runoff that is eventually integrated into the isotopic composition of the lake. These inter-related aspects will be subject to climatic variation responses across the lake catchment system, possibly offsetting warming effects as mediated by evaporation and altering the deviation between theoretical slopes of LEL predicted by Craig and Gordon^11,27^ versus those found in the lake data. We also found the influence of surface energy parameters like the Bowen ratio and solar radiation on the overall lake isotopic composition became even more important when the lake size was considered.

Our study indicates that the stable water isotopes of global lakes are highly relevant indicators that integrate multiple processes at the watershed scale and are sensitive to the hydroclimate response of both lakes and their catchment systems. Stable isotope assays provide a low-cost efficacious tool to study lake-catchment changes with regards to sample collection and isotopic analysis. Additionally, stable isotope data from lakes are fully comparative globally, thereby providing a competitive advantage under the current scenario of different international methods and approaches that are not easy to compare in time and scale and which result in the current lack of the comparable data for lakes and catchments^32,33^. Our results showed that together with key parameters like lake surface temperature, water level, ice cover, and lake color, stable isotopes might be considered as essential climate response variables that will contribute critically to the characterization of the hydrological cycle and better prediction of lake responses to climatic variability and ecosystem changes.

Compared to remote sensing-based assessments of changes in global lakes, stable isotope variations directly reflect all of the phase changes and mixing that occurs as water passes through the entire catchment-lake hydrological cycle, thereby integrating regional and local in situ hydroclimate conditions and distinctions that may not be evident from satellite observations^15,19^. Accordingly, as a natural hydrological tracer, long-term stable water isotope monitoring of Earth’s lakes complements and contributes to validation and fine-tuning of satellite-based approaches and by providing explicit data about their evaporation status.

## Methods

The global lake water isotope data is comprised of unpublished data from the International Atomic Energy Agency (IAEA) and from the scientific literature. Catchment polygons were delineated using QGIS v3.16 and a hydrologically conditioned raster digital elevation mode (DEM); see the “Methods” online. For detailed descriptions of the data sources and the treatment of environmental data in RCWIP2, WorldClim2, GLDAS Noah Land Surface Model, CGIAR-CSI, and HydroATLAS (2019) the reader is referred to the Methods online. All lake *δ*^18^O and *δ*^2^H data were normalized to the isotopic composition of each lake’s catchment precipitation amount-weighted inputs^34^, which was defined as the difference between isotope value in the lake (*δ*^18^O_L_ or *δ*^2^H_L_) and that of precipitation (*δ*^18^O_P_ or *δ*^2^H_P_) falling on its catchment (Eq. 1):

1 $${\Delta }_{{{{{{\rm{L}}}}}}-{{{{{\rm{P}}}}}}}{\delta }^{18}{{{{{\rm{O}}}}}}({\varDelta }_{{{{{{\rm{L}}}}}}-{{{{{\rm{P}}}}}}}{\delta }^{2}{{{{{\rm{H}}}}}})={\delta }^{18}{{{{{{\rm{O}}}}}}}_{{{{{{\rm{L}}}}}}}({\delta }^{2}{{{{{{\rm{H}}}}}}}_{{{{{{\rm{L}}}}}}})-{\delta }^{18}{{{{{{\rm{O}}}}}}}_{{{{{{\rm{P}}}}}}}({\delta }^{2}{{{{{{\rm{H}}}}}}}_{{{{{{\rm{P}}}}}}})$$

These normalized data (Δ_L-P_*δ*^18^O and Δ_L-P_*δ*^2^H) were referenced as evaporative enrichment^14^ and were converted to median values per lake in the case of lakes with multiple data points. Non-normalized lake isotope data were used for estimations of the evaporation to inflow ratios (E/I) using a lake catchment isotope-mass balance model^10,11,35^ based on the isotopic equilibrium separation^36^ and the isotopic composition of the evaporation flux^11,35^. The E/I result per lake was accepted only if there was an acceptable agreement between the O and H isotope results. A gaussian mixture model based on expectation maximization (EM) and Bayesian information criterion (BIC) was used to cluster the lakes according to their determined E/I rate (XLSTAT Basic+, v.2021.2).

One-way ANOVA was used to determine the significance of the lake’s local evaporation line (LEL) slope and intercept (the 95% confidence level with a *p*-value < 0.05). We used random forest regression with bootstrapped aggregating to estimate Δ_L-P_*δ*^18^O and Δ_L-P_*δ*^2^H as a function of various predictors (R package ‘randomForest’)^37^. Estimation of the model error was obtained by leveraging the out-of-bag (OOB) and the mean square error (MSE in %). The meaningfulness of each predictor variable was tested using MSE, where predictors that showed the highest MSE were considered as important to model the target variable of lake *δ* value, whereas predictors with low MSE were considered as unimportant. The residual sum of squares (*R*^*2*^) was used to indicate the model ability to capture the % of overall variability. A detailed description of the methods and data availability along with associated codes and references are available in the Methods online.

## Supplementary information

Supplementary Information

Peer Review File

Description of Additional Supplementary Files

Supplementary Data 1

## Data Availability

The data on 1264 lakes generated in this study are provided in the Supplementary Information (Supplementary Data 1). The additional data can be obtained by request at https://nucleus-new.iaea.org/sites/ihn/Pages/GNIR.aspx.

## Code availability

All code sources used in the study are mentioned with original references and available for public.

## References

[1] Woolway RI (2020). Global lake responses to climate change. Nat. Rev. Earth Environ..

[2] Wang W (2018). Global lake evaporation accelerated by changes in surface energy allocation in a warmer climate. Nat. Geosci..

[3] Brutsaert W, Parlange MB (1998). Hydrologic cycle explains the evaporation paradox. Nature.

[4] Zhao G, Gao H, Cai X (2020). Estimating lake temperature profile and evaporation losses by leveraging MODIS LST data. Remote Sens. Environ..

[5] Priestley CHB, Taylor RJ (1972). On the assessment of surface heat flux and evaporation using large-scale parameters. Mon. Weather Rev..

[6] Fritschen LJ (1965). Accuracy of evapotranspiration determinations by the Bowen ratio method. Hydrol. Sci. J..

[7] Walter, I. A. et al. In *Watershed Management and Operations Management 2000* 1−11 (American Society of Civil Engineers (ASCE), 2000).

[8] Gonfiantini R, Wassenaar LI, Araguas-Araguas L, Aggarwal PK (2018). A unified Craig-Gordon isotope model of stable hydrogen and oxygen isotope fractionation during fresh or saltwater evaporation. Geochim. Cosmochim. Acta.

[9] Vystavna Y, Matiatos I, Wassenaar LI (2020). 60-year trends of δ^18^O in global precipitation reveal large scale hydroclimatic variations. Glob. Planet. Change.

[10] Gibson JJ, Birks SJ, Yi Y (2016). Stable isotope mass balance of lakes: a contemporary perspective. Quat. Sci. Rev..

[11] Craig, H. & Gordon, L. I. *Deuterium and Oxygen 18 Variations in the Ocean and the Marine Atmosphere* (1965).

[12] Terzer S, Wassenaar LI, Araguás-Araguás LJ, Aggarwal PK (2013). Global isoscapes for δ^18^O and δ^2^H in precipitation: improved prediction using regionalized climatic regression models. Hydrol. Earth Syst. Sci..

[13] Halder J, Terzer S, Wassenaar LI, Araguas-Araguas LJ, Aggarwal PK (2015). The Global Network of Isotopes in Rivers (GNIR): integration of water isotopes in watershed observation and riverine research. Hydrol. Earth Syst. Sci..

[14] Gibson JJ, Birks SJ, Edwards TWD (2008). Global prediction of δ_A_ and δ^2^H-δ^18^O evaporation slopes for lakes and soil water accounting for seasonality. Glob. Biogeochem. Cycles.

[15] Jasechko S (2013). Terrestrial water fluxes dominated by transpiration. Nat. Commun..

[16] Peel MC, Finlayson BL, McMahon TA (2007). Updated world map of the Koppen−Geiger climate classification. Hydrol. Earth Syst. Sci..

[17] Kottek M (2006). World map of the Köppen−Geiger climate classification updated. Meteorologische Z..

[18] Kebede S, Travi Y, Rozanski K (2009). The δ^18^O and δ^2^H enrichment of Ethiopian lakes. J. Hydrol..

[19] Gibson JJ, Bursey GG, Prowse ТО (1993). Estimating evaporation using stable isotopes: quantitative results and sensitivity analysis. Nord. Hydrol..

[20] Dansgaard W (1964). Stable isotopes in precipitation. Tellus.

[21] Jasechko S (2019). Global isotope hydrogeology—review. Rev. Geophys..

[22] Gibson JJ (2001). Forest-tundra water balance signals traced by isotopic enrichment in lakes. J. Hydrol..

[23] Brooks JR (2018). Estimating wetland connectivity to streams in the Prairie Pothole Region: an isotopic and Remotes sensing approach. Water Resour. Res..

[24] HydroAtlas v.1. A global compendium of hydro-environmental sub-basin and river reach characteristics at 15 arc-second resolution. (ed. Lehner, B.) https://www.hydrosheds.org/images/inpages/HydroATLAS_TechDoc_v10.pdf (2019). 10.1038/s41597-019-0300-6 PMC6901482 31819059

[25] Ala-Aho P (2018). Permafrost and lakes control river isotope composition across a boreal Arctic transect in the Western Siberian lowlands. Environ. Res. Lett..

[26] Woo Ming‐ko (1986). Permafrost hydrology in North America. Atmosphere-Ocean.

[27] Craig H, Gordon L, Horibe Y (1963). Isotopic exchange effects in the evaporation of water: 1. Low-temperature experimental results. J. Geophys. Res..

[28] Vallet-Colomb C, Gasse F, Sonzogni C (2008). Seasonal evolution of the isotopic composition of atmospheric water vapour above a tropical lake: deuterium excess and implication for water recycling. Geochim. Cosmochim. Acta.

[29] Bowen GJ (2018). Inferring the source of evaporated waters using stable H and O isotopes. Oecologia.

[30] Gonfiantini R, Wassenaar LI, Araguas-Araguas L (2020). Stable isotope fractionations in the evaporation of water: the wind effect. Hydrol. Process..

[31] Gonfiantini R (2001). The altitude effect on the isotopic composition of tropical rains. Chem. Geol..

[32] Vinma LR, Medhaug I, Schmidt M, Bouffard D (2021). The vulnerability of lakes to climate change along an altitudinal gradient. Commun. Earth Environ..

[33] UNEP. *UNEP Frontiers 2016 Report: Emerging Issues of Environmental Concern* (United Nations Environment Programme, 2016).

[34] Terzer-Wassmuth S, Wassenaar LI, Welker J, Araguas-Araguas LJ (2021). Improved high‐resolution global and regionalized isoscapes of δ^18^O, δ^2^H and d-excess in precipitation. Hydrol. Process.

[35] Gonfiantini, R. In *Handbook of Environmental Isotope Geochemistry.**The Terrestrial Environment B* (eds Fritz, P., Fontes, J. C.) 113−168 (Elsevier, 1986).

[36] Horita J, Wesolowski DJ (1994). Liquid-vapor fractionation of oxygen and hydrogen isotopes of water from the freezing to the critical temperature. Geochim. Cosmochim. Acta.

[37] Liaw, A. & Wiener, M. Classification and regression by randomForest. R. N. 2, 18–22 (2002).

