# Supplementary material for: Stable isotopes in global lakes integrate catchment and climatic controls on evaporation
Source: Nat Commun. 2021 Dec 10;12:7224. doi: 10.1038/s41467-021-27569-x (PMC8664878; doi:10.1038/s41467-021-27569-x)
Supplement: Supplementary file 1 — Supplementary Information [file 41467_2021_27569_MOESM1_ESM.pdf]

Supplementary information

Stable isotopes in global lakes integrate catchment and climatic controls on evaporation

Yuliya VYSTAVNA[1]*, Astrid HARJUNG[1], Lucilena R. MONTEIRO[1], Ioannis MATIATOS[1], and Leonard I. WASSENAAR[1]

[1]International Atomic Energy Agency, Isotope Hydrology Section, Vienna International Centre, A-1400, Vienna, Austria

*Corresponding Author: y.vystavna@iaea.org

Contents

1. Supplementary Figures 2

2. Supplementary Methods 7

Spatial distribution of global lakes 7

Statistical treatment and data normalization 8

Random forest model 9

Isotope mass balance modelling (E/I) 14

References 15

1. Supplementary Figures

The δ^2H_L (despite fewer data then ^{18}O) had comparable spatial patterns in global lakes with the $\delta^{18}O_L$ values in respect to the climate zone (Figure S1).

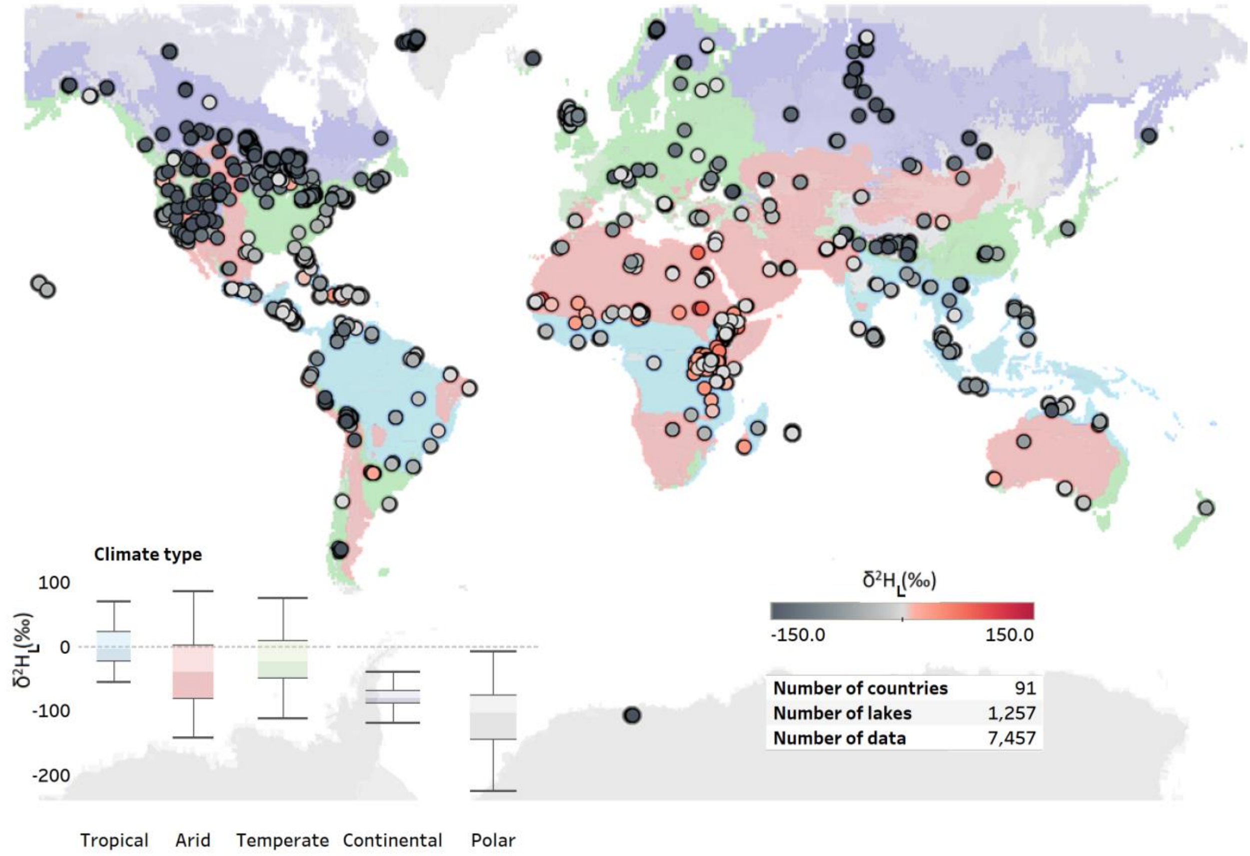

Figure S1. Distribution of the δ^2H_L composition of global lakes by climatic zone (Köppen-Geiger). Median and ranges of δ^2H_L values in lakes by climate zones are depicted in the box-and-whiskers plots. The climate zone map was generated according to Kottek et al. (2006)[1].

The latitudinal gradient relationship in lake isotopic composition was mirrored in climate factors including surface albedo, solar radiation and air temperature distribution patterns. Unlike for precipitation, there was no isotopic pattern or correlation of the lake's isotopic composition to its altitude (Figure S2).

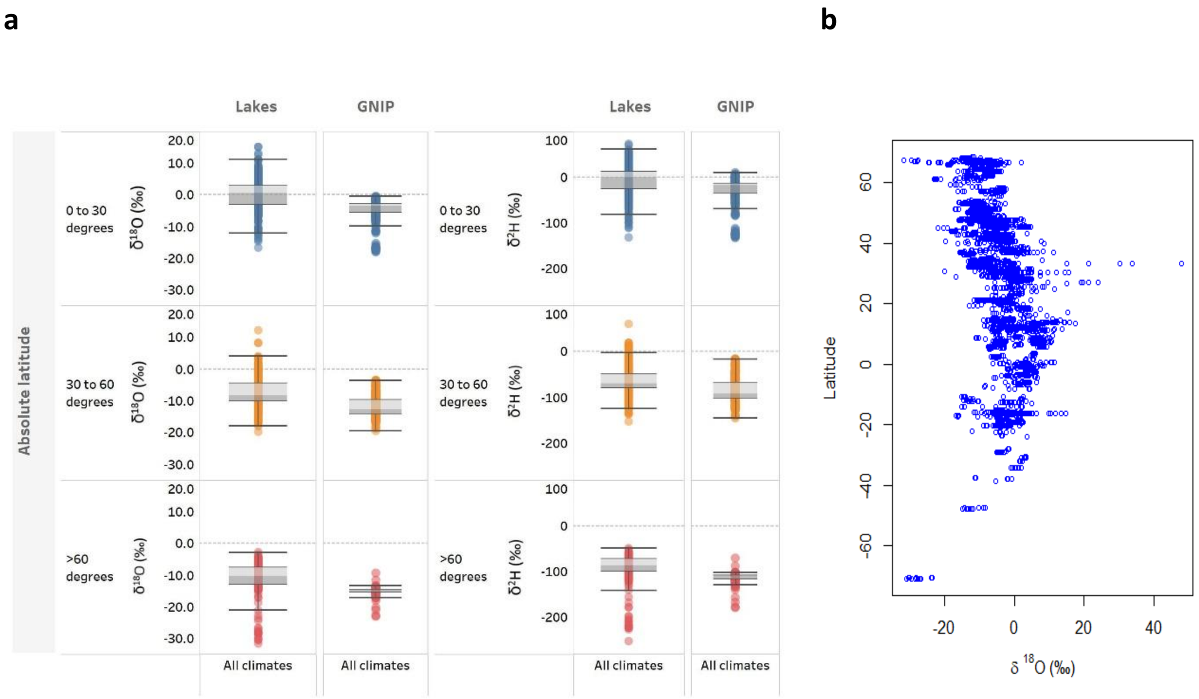

Figure S2. Latitudinal gradient of the δ^{18}O and δ^{2}H in global precipitation (GNIP) and lakes in relation to the isotope composition in precipitation (a) and along the overall latitudinal gradient (b)

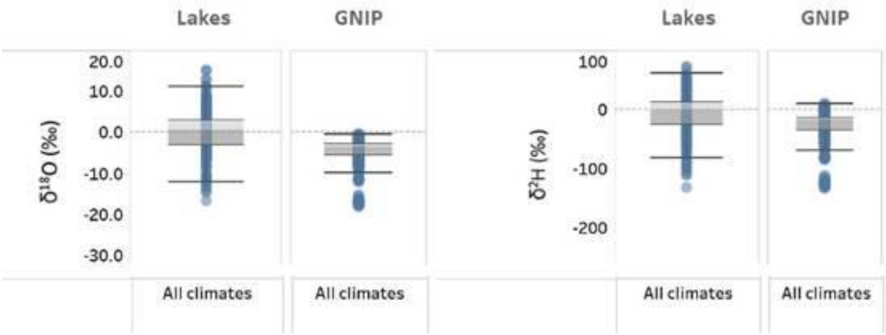

Figure S3. The boxplots show the difference in the isotopic composition of lakes and catchment-weighted precipitation (t-test results: p-value < 0.0001).

Highly-sensitive-to-evaporation-lakes were all characterized by having the highest isotopic and evaporative enrichment δ values (Figure S4).

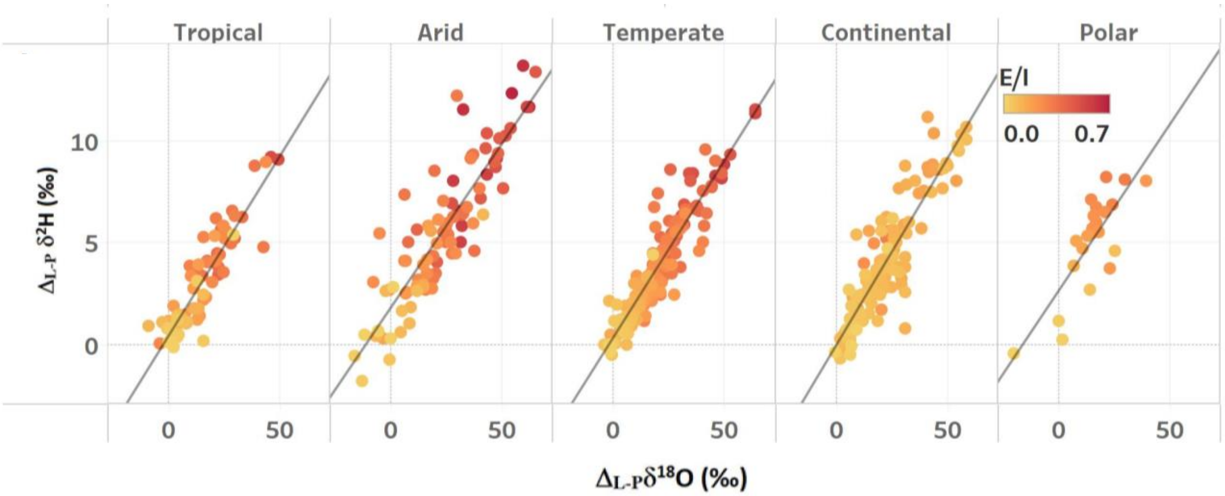

Figure S4. Regression of the evaporative enrichment $\Delta_{L\text{-}P}\delta^2H$ versus $\Delta_{L\text{-}P}\delta^{18}O$ plot in relation to the E/I rate

Table S1. Catchments and isotopic characteristics of lakes in relation to E/I

E/I	Catchment area (km^2)	Lake area (km^2)	ET (mm)	Precipitation (mm)	Lake $\delta^{18}O$	Lake δ^2H
>0.4	1082.5	24.0	1737.0	735.2	-0.9	2.7
0.2-0.4	160.5	3.2	1671.1	1066.7	-1.7	-10.9
<0.2	61	0.4	748	807	-8	-78

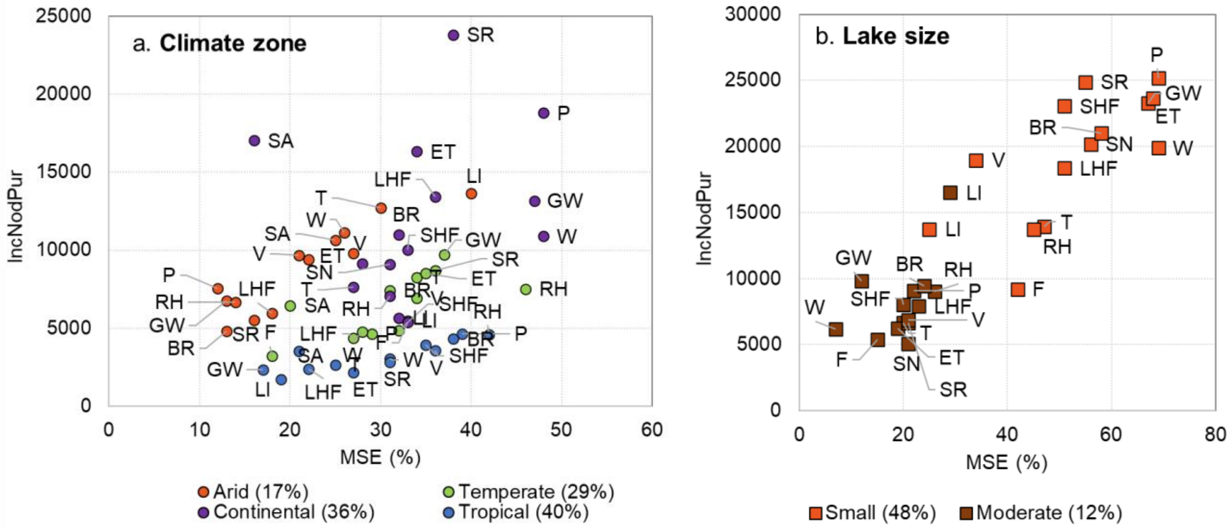

Figure S5. Random forest model results of variable control on the evaporative enrichment ($\Delta_{L\text{-}P}\delta^2$H) in lakes in respect to the climatic zone (a) and lake size (b). Physical meaningfulness of the variables was tested using the Mean Decrease Accuracy (MSE, %) that shows how much model accuracy decreases in case of elimination of the variable and the Mean Decrease Gini (IncNodPur) that is a measure of variable importance based on the Gini impurity index. Environmental variables used in the random forest: Bowen ratio (BR, dimensionless), evapotranspiration (ET, mm), forest coverage in the catchment (F, %), groundwater table (G, cm), catchment limnicity (LI, %), latent heat fluxes (LHF, W/m^2), precipitation amount (P, mm), relative humidity (RH, %), lake surface area (SA, km^2), sensitive heat fluxes (SHF, W/m^2), snow coverage in the catchment (SN, %), solar radiation (SR, kJ/m^2), air temperature (T, °C), Vapor saturation (V, kPa) and wind speed (W, m/s). Model performance (R^2) is presented in % in the legend.

2. Supplementary Methods

Spatial distribution of global lakes

The isotope dataset was biased by higher amount (> 80%) of lakes from the mid and high latitudes (northern of 40° N and southern of 40° S). Few lakes were in the polar climates. Additionally, small lakes (< 10 km^2 of the surface area) represented > 50% of the lakes and most of them were in the continental climate zone. These patterns follow the natural distribution of global lakes[2,3]. Isotope data were obtained from the project archive of the Isotope Hydrology Section and from published studies. In the database, 12% of lakes were artificial, and created by the damming of the rivers. The database covers the period 1956 to 2020, where most lakes (64%) were sampled sporadically from 1990 to 2020. Only 15 out of 1,257 lakes were sampled systematically for 5-7 years on average, which did not allow us to investigate the time trends. Like in other studies[4-6], we assumed that lakes operate close to their hydrological and isotopic steady states attained during their long history[7] with low interannual variation in the time frame covered in the study.

The global lakes dataset is available online at https://nucleus.iaea.org/sites/ihn .

Lake grouping by size was done according to the classification of M. Meybeck[3], where small lakes were those with a surface area of < 10 km^2, moderate (medium) lakes have a surface area range from 10 to 500 km^2 and large (great) lakes were those with the surface area of > 500 km^2.

According to the salinity level, the lakes were divided in two groups: 1) hypersaline lake (the lake water surpass the salinity of the ocean water – 35 g/L) and 2) non hypersaline lakes[8].

To generate the map (Figure 1), we used R script, provided as an open access source at http://koeppen-geiger.vu-wien.ac.at/present.htm#GoogleEarth[1]. The R script was published under the: Creative Commons CC-BY-NC license (see: https://creativecommons.org/licenses/by-nc-sa/3.0/).

Statistical treatment and data normalization

Land surface and subsurface runoff and direct precipitation to the lake affect the isotope composition and can offset the effects of evaporation. To remove the impact of modern precipitation on the isotopic composition of the lake, we normalized our data of the median isotopic composition of lake ($\delta^{18}O_L$ or δ^2H_L) to the isotopic composition of catchment amount-weighted precipitation ($\delta^{18}O_P$ or δ^2H_P) by:

$$\Delta_{L\text{-}P}\delta^{18}O\ (\Delta_{L\text{-}P}\delta^2H) = \delta^{18}O_L\ (\delta^2H_L) - \delta^{18}O_P\ (\delta^2H_P) \qquad (1)$$

The $\Delta_{L\text{-}P}\delta^{18}O$ ($\Delta_{L\text{-}P}\delta^2H$) values are referred as the evaporative enrichment.

Evaporative enrichment values used were the lake median and evaluated for normality using the Kolmogorov-Smirnov and Anderson-Darling normality tests.

Two ANOVA models were used: Model 1 is $\Delta_{L\text{-}P}\delta^2H \sim \Delta_{L\text{-}P}\delta^{18}O$ and Model 2 is $\Delta_{L\text{-}P}\delta^2H \sim \Delta_{L\text{-}P}\delta^{18}O$ * Climate. Akaike information criterion (AIC) was used to check goodness of fit of statistical models. Results showed that Model 2 was better in goodness of fit (lower AIC) than Model 1 and indicated that the Climate was a significantly important variable.

Ordinary Least Square Regression (OLS), Moving Average (MA), Standardized Major Axis (SMA) and Reduced Major Axis (RMA) regression models were applied for $\Delta_{L\text{-}P}\delta^2H \sim \Delta_{L\text{-}P}\delta^{18}O$ and $\delta^2H \sim \delta^{18}O$ regression lines considering several confidence intervals for intercept and slope (2.5%-Intercept, 97.5%-Intercept, 2.5%-Slope and 97.5%-Slope). All models were tested and OLS

showed the best fit and lowest residue value on the raw and lake median values, and hence were used to plot the regression lines in the study. $\Delta_{L\text{-}P}\delta^2H$ ~ $\Delta_{L\text{-}P}\delta^{18}O$ regression line is referred to the 'local evaporation line' (LEL)[9]. The intersect was defined as the crossing point of the LEL with the Global meteoric water line (GMWL)[9-11]. This intersection represents the estimated isotopic value of water input to the lake before the evaporation. The universal GMWL as $\delta^2H = 8\ \delta^{18}O+10$ was used due to the theoretical background[9,12].

Random forest model

The selection of the best random forest (RF) method (supervised machine-learning tool) was based on the proven applicability of the RF method in hydrological, hydrogeological and isotopic studies[13-15]. RF is both a regression and classification tool that is promoted by deep learning, available programming packages and applied to a growing amount of environmental data.

For RF simulation, we used 'randomForest' package in R[16]. The evaporative enrichment ($\Delta\delta^{18}O_{L\text{-}P}$ and $\Delta\delta^2H_{L\text{-}P}$) was used as a primary factor and the sets of variables (Table S2) was used as explanatory variables. The model was trained using a different number of trees (from 1,000 to 5,000) and 4,000 trees were selected for the final modelling. The selection was based on the stability of the out of bag value (OOB) and the general model performance (regression coefficient, R^2). Physical meaningfulness of the explanatory variables in RF model was tested using the Mean Decrease Accuracy (%IncMSE) that shows how much model accuracy decreases in case of elimination of the variable and the Mean Decrease Gini (IncNodePurity) that is a measure of variable importance based on the Gini impurity index used for the calculating the splits in trees.

The higher the value of mean decrease accuracy or mean decrease Gini score, the higher the importance of the variable to the model.

Table S2. Primary and the explanatory values (EV) used in the Random Forest model

Factors used in the Random Forest		Abbreviation	Factor value	Data origin
Primary Factor	Evaporative enrichment	$\Delta_{L\text{-}P}\delta^{18}O$ ($\Delta_{L\text{-}P}\delta^{2}H$)	‰	Calculated as the difference between the isotopic composition of lake and precipitation. The precipitation amount weighted isotopic values were obtained from Terzer-Wassmuth et. (2021)[17].
EV	Air temperature	T	°C	Catchment weighted values were retrieved from Fick & Hijmans (2017)[18].
EV	Precipitation amount	P	mm/yr	Catchment weighted values were retrieved from Fick & Hijmans (2017)[18].
EV	Wind speed	W	m/s	Catchment weighted values were retrieved from Fick & Hijmans (2017)[18].
EV	Solar radiation	S	kJ/ m^2d	Catchment weighted values were retrieved from Fick & Hijmans (2017)[18].
EV	Vapor pressure	V	kPa	Catchment weighted values were retrieved from Fick & Hijmans (2017)[18].
EV	Relative humidity	RH	%	Calculated by dividing V with saturated vapor pressure (calculated from T). Catchment weighted values of V & T were retrieved from Trabucco & Zomer (2019)[19].
EV	Evapotranspiration	ET	mm/year	Evapotranspiration calculated by Penman Monteith method taken from Trabucco & Zomer (2019)[19].
EV	Latent heat fluxes	LHF	W/m^2	Latent heat flux is the flux of heat from the Earth's surface to the atmosphere that is associated with evaporation of water at the surface and subsequent condensation of water vapor in the troposphere (www.unescwa.org). Catchment weighted values were retrieved and averaged from GLDAS Noah Land

				Surface Model L4 monthly 0.25 x 0.25 degree V2.1[20]. GLDAS – the Global Land Data Assimilation system is described in Rodell et al. (2004)[21].
EV	Sensible heat fluxes	SHF	W/m^2	The sensible heat flux is the transfer of heat caused by the difference in temperature between the sea and the air[22]. Catchment weighted values were retrieved and averaged from GLDAS Noah Land Surface Model L4 monthly 0.25 x 0.25 degree V2.1[20]. GLDAS – the Global Land Data Assimilation system is described in Rodell et al. (2004)[21].
EV	Bowen ratio	BR	dimensionless	The ratio of heat losses by conduction and by evaporation from any water surface, calculated by I. S. Bowen[23]. Calculated by dividing sensible heat flux by latent heat flux
EV	Limnicity	Limn	10x %Lake area in the catchment	Lake area density (calculated as percent area covered by lakes within a 25 km radius)[7].
EV	Snow cover extent	Snow	%	Snow cover extent shows the share of the catchment area that is permanently or seasonally covered with snow and is derived from the daily global sunlit images for the period between July 2002 and April 2015 [24].
EV	Groundwater table depth	GWtab	cm	Data obtained from HydroAtlas (2019)[24] with the reference on Fan et al. (2013)[25].
EV	Forest cover extent	Forest	%	Forest cover extent shows the share of the catchment area that is covered with forest (GLC2000 land cover map in HydroAtlas 2019)[24]
EV	Surface area	Sarea	km^2	Obtained from different sources, when available from the studies and projects or obtained from Google Earth.

Catchments were delineated using QGIS v3.16 and hydrologically conditioned raster DEM (3 arc-second spatial resolution) from the hydroSHEDS project below 55°[26]. Above 55° we used the raster DEM provided by the NASA Shuttle Radar Topography Mission (SRTM) downloader

plug-in from the USGS if available or downloaded the GMTED2010 DEM from the USGS Earth Explorer Website with 30 Arc-second spatial resolution. For some small lakes <0.1km^2 in Greenland, Canada, Finland and Russia this resolution was too low to get a realistic catchment estimation based on elevation data. After obtaining all the individual catchments of the lakes as a shape file we performed zonal statistics of the following raster input layers: air temperature, precipitation amount, wind speed, solar radiation, relative humidity, latent heat fluxes, sensible heat fluxes, vapor pressure and Bowen ratio. Catchment weighted data on limnicity, snow cover, groundwater table level and forest cover were obtained from HydroAtlas v. 1 (2019)[20] (Table).

Seasonal effect was eliminated by the conversion of raw isotope values in lake median value and so season was not considered in the model. Additionally, considering that all lake systems undergo short term fluctuation caused by the seasonality of individual hydrological components (precipitation, inflow, lake water)[27]. Isotopic seasonality was considered in our study based on the following assumptions and calculations: (i) groundwater that is often the dominant inflow component in the lake was assumed to have no seasonal variations. The assumption is based on previous studies that showed that groundwater has water transit time more than 1 year and biases the seasonality of precipitation[28], (ii) precipitation isotopic seasonality was removed by the normalization of the isotopic signation of the lake by the catchment weighted precipitation that refer to the 'evaporative enrichment' ($\Delta_{L\text{-}P}\delta^{18}O$), (iii) according to the sampling method[27], sampling for isotope analysis should be done during the mixing period or the lake water profile should be sampled and converted to the average that represents the mixing conditions or alternatively, lake outflow should be sampled. Therefore, we assumed sampling was done according to these guidelines and the isotopic stratification of the lake column was integrated in

the lake isotopic fingerprint. It is also assumed that lakes were sampled during the evaporation period which is the reason for conducting isotope mass balance studies [10,11,27].

Variables like lake depth, water residence time and stratification were excluded from the modelling due to a lack of accurate or available data for more than 50% of lakes and potentials strong variation. No significant difference ($p < 0.05$) was found between the evaporative enrichment ($\Delta_{L\text{-}P}\delta^{18}O$ vs $\Delta_{L\text{-}P}\delta^{2}H$) slopes regarding the salinity and hence salinity was excluded from the explanatory variables. Altitude, latitude, longitude, and catchment area were excluded from the RF as these parameters were used to obtain the catchment-weighted evaporative enrichment and catchment weighted isotopic composition of precipitation. Wetland and soil water were not included in the explanatory variables because these variables are covered under the limnicity variable. Lake surface temperature (LST) was not included in the RF because we were able to retrieve LST data only for ca. 30% of lakes from our database and these were strongly biased to large lakes. Also, classical isotope-mass balance modelling approach based on the assumption that the mean annual temperature of the lake is identical to the mean annual surface air temperature in the area[10,27,28]. The correlation between retrieved lake surface temperature and air temperature was high (R^2=0.61) that confirm the reliability of the undertaken assumption. While the temperature of lake can be different from the air, it is likely biased at the annual average scale that is considered in this study.

Snow, permafrost and glacier variables were considered in continental and polar climates only. While snow cover was expected in some temperate and even arid areas, this parameter decreased the RF model performance and was eliminated in the final stages of assessment. The same approach was used for forest cover which was included in the RF of temperate and

continental lakes. The RF results had low significant ($R^2 < 10\%$) for polar and great lakes and were excluded in the paper.

Isotope mass balance modelling (E/I)

We determined the evaporation/input (E/I) ratios for a subset of 548 lakes that had sufficient data. Whenever the Isotope-enabled model showed the high discrepancy in E/I between the O and H two isotopes (i.e. > 20% discrepancy), the lake was considered isotopically unbalanced. Additionally, some lakes could not be considered because only $\delta^{18}O$ was available for the water balance calculations, or the model could not be appropriately applied (i.e., deep groundwater recharge). We assumed hydrological steady-state conditions in the lake that are mainly observed during the mixing period and the isotopic mass balance during this period were estimated [10,11] as:

$$I\delta_I = Q\,\delta_Q + E\,\delta_E \qquad (2)$$

where I (m^3/yr) is total water inflow into the lake, including precipitation, surface and subsurface flows, E is the evaporation flux, Q (m^3/yr) is the lake outflow, and δ_I and δ_Q (‰) are the amount-weighted (AW) mean isotopic compositions of total inflow and outflow. The isotopic composition of the evaporation flux (δ_E, ‰) was estimated according to the model proposed by Craig and Gordon (1965)[9] and Gonfiantini (1986)[29] as:

$$\delta_E = \left(\frac{\delta_L - \varepsilon^+}{\alpha^+} - \mathrm{h}\delta_A - \varepsilon_k\right)/(1 - \mathrm{h} + \varepsilon_k 10^{-3}) \qquad (3)$$

where the isotopic composition of atmospheric moisture δ_A was calculated based on an equilibrium approach[10,11]. The relative humidity (h) was obtained from Fick & Hijams (2017)[18] and was a dimensionless parameter. The isotopic separation equilibrium (ε^+) was calculated based on the isotopic fractionation (α^+)[30]. The kinetic isotopic separation (ε_k) was calculated based on the

relative humidity, transport resistant parameters (equal to one when the evaporation rate is controlled by molecular transport of water through the laminar layer to the atmosphere) and a kinetic constant, similar to the approach described by Gibson et al. (2016a,b)[10,11].

The evaporation to inflow ratio (E/I) of the lake was calculated according to Gibson et al (2016a,b)[10,11] as:

$$\frac{E}{I} = \frac{(\delta_I - \delta_Q)}{(\delta_E - \delta_Q)} \qquad (4)$$

A Gaussian mixing model (GMM) was used to identify E/I categories. GMM is a probabilistic model that assumes all the data points are generated from a mixture of a finite number of Gaussian distributions with unknown parameters. For the modelling, we used expectation-minimization (EM) algorithm for fitting the mixture. Bayesian Information Criterion (BIC) was used to assess the number of clusters in the data[31]. Based on the modelling results, three lakes groups according to E/I value were determined: (i) < 0.2 (lakes with low evaporation losses); (ii) 0.2 – 0.4 (lakes with moderate evaporation losses) and (iii) > 0.4 (lakes with high evaporation losses).

References

1. Kottek, M. et al. World Map of the Köppen-Geiger climate classification updated. *Meteorologische Zeitschrift* **15**, 259-263 (2006)
2. Wang, W. et al. Global lake evaporation accelerated by changes in surface energy allocation in a warmer climate. *Nat. Geosci.* **11**, 410 (2018).
3. Meybeck, M. Global Distribution of Lakes. In: Lerman A., Imboden D.M., Gat J.R. (eds) Physics and Chemistry of Lakes. Springer, Berlin, Heidelberg. https://doi.org/10.1007/978-3-642-85132-2_1 (1995).
4. Jasechko, S. et al. Terrestrial water fluxes dominated by transpiration. Nature Commun. **496**, 347-350 (2013).
5. Gibson, J.J., & Reid, R. Water balance along a chain of tundra lakes: A 20-year isotopic perspective. *J. Hydrol.* **519**, 2148-2164 (2014).

6. Isokangas, E., Rozanski, K., Rossi, P.M., Ronkanen, A.-K. & Klove B. Quantifying groundwater dependence of a sub-polar lake cluster in Finland using an isotope mass balance approach. *Hydrol. Earth Sys. Sci.* **19**, 1247-1262 (2015).
7. Messager, M. et al. Estimating the volume and age of water stored in global lakes using a geo-statistical approach. *Nat. Commun*. **7,** 13603 (2016).
8. Thorslund, J. & van Vliet, M.T.U. A global dataset of surface water and groundwater salinity measurements from 1980-2019. *Sci. Data* **7**, 231 (2020).
9. Craig, H. & Gordon, L. I. Deuterium and oxygen 18 variations in the ocean and the marine atmosphere (1965).
10. Gibson, J.J. et al. Stable isotope mass balance of fifty lakes in central Alberta: Assessing the role of water balance parameters in determining trophic status and lake level. *Journal of Hydrology: Regional Studies* **6**, 13-25 (2016a).
11. Gibson, J. J., Birks, S. J. & Yi, Y. Stable isotope mass balance of lakes: a contemporary perspective. *Quat. Sci. Rev.* **131**, 316-328 (2016b).
12. Gat, J.R. Oxygen and hydrogen isotopes in the hydrological cycle. *Annu. Rev. Earth Planet. Sci.* **24 (1)**, 225-262 (1996).
13. Koch, J. *et al*. Modeling depth of the redox interface of high resolution at national scale using random forest and residual gaussian stimulation. *Water Resource Research* **55 (2)**, 1451-1469 (2019).
14. Hajek, M. et al. 2021. A European map of pH and calcium in groundwater. *Earth Syst. Sci. Data* **13**, 1089–1105 (2021).
15. Oczkowski, A., Kreakie, B., McKinney, R.A., Prezioso, J. Patterns in Stable Isotope Values of Nitrogen and Carbon in Particulate Matter from the Northwest Atlantic Continental Shelf, from the Gulf of Maine to Cape Hatteras. *Front. Mar. Sci.* **3**, 252-260 (2016).
16. Liaw, A. & Wiener, M. Classification and Regression by randomForest. *R News* **2(3)**, 18—22 (2002).
17. Terzer-Wassmuth, S., Wassenaar, L. I., Welker, J. & Araguas-Araguas, L. J. New High-Resolution Global and Regionalized Isoscapes of δ^{18}O, δ^{2}H, and d-Excess in Precipitation. *Hydrol. Process.* **in press** (2021).
18. Fick, S.E. & Hijmans, R.J. WorldClim 2: new 1km spatial resolution climate surfaces for global land areas. *Int. J. Climatol.* **37 (12)**, 4302-4315 (2017).
19. Trabucco, A. & Zomer, R. Global Aridity Index and Potential Evapotranspiration (ET0) Climate Database v2. figshare. Fileset. https://doi.org/10.6084/m9.figshare.7504448.v3 (2019).
20. Beaudoing, H. & Rodell, M. NASA/GSFC/HSL (2020); GLDAS Noah Land Surface Model L4 monthly 0.25 x 0.25 degree V2.1, Greenbelt, Maryland, USA, Goddard Earth Sciences Data and Information Services Center (GES DISC), Accessed on: 2021-02-11, 10.5067/SXAVCZFAQLNO (2020).
21. Rodell, M., et al. The Global Land Data Assimilation System, *Bull. Amer. Meteor. Soc.* **85**, 381-394, (2004).
22. Encyclopedia of Ocean Sciences. Eds. by J. Kirk Cochran, Henry J. Bokuniewicz & Patricia L. Yager. Elsevier (2019).
23. Bowen, I.S. The ratio of heat losses by conduction and by evaporation from any water surface. Phys. Rev. 27 (6), 779-787 (1926).
24. HydroAtlas v.1, 2019. A global compendium of hydro-environmental sub-basin and river reach characteristics at 15 arc-second resolution. Editor: Bernhard Lehner (2019).
25. Fan, Y., Li, H., & Miguez-Macho, G. Global patterns of groundwater table depth. Science **339**(6122), 940-943 (2013).
26. Lehner, B. & Grill, G. Global river hydrography and network routing: baseline data and new approaches to study the world's large river systems. *Hydrol. Process.* 27(15): 2171–2186. Data is available at www.hydrosheds.org. (2013).

27. Rozanski, K., Froehlich, K., Mook, W.G. Environmental isotopes in the hydrological cycle. Principles and Applications 39, Volume III. Surface water. Technical documents in hydrology. Edited by W.G. Mook. Published by UNESCO (2001).
28. Jasechko, S. Global isotope hydrogeology—review. *Rev. Geophys*. **835-965**, 57 (2019).
29. Gonfiantini, R., 1986. Chapter 3–Environmental Isotopes in Lake Studies. Editor(s): P. Fritz, J.Ch. Fontes. In Handbook of Environmental Isotope Geochemistry. The Terrestrial Environment B. Elsevier, pp. 113-168 (1986).
30. Horita, J. & Wesolowski, D.J. Liquid-vapor fractionation of oxygen and hydrogen isotopes of water from the freezing to the critical temperature. *Geochim. Cosmochim. Acta* **58 (16)**, 3425-3437 (1994).
31. Reynolds, D. Gaussian Mixture Models. In: Li S.Z., Jain A. (eds) Encyclopedia of Biometrics. Springer, Boston, MA. https://doi.org/10.1007/978-0-387-73003-5_196 (2009).
