# Supplementary material for: Stable isotopes in global lakes integrate catchment and climatic controls on evaporation
Source: Nat Commun. 2021 Dec 10;12:7224. doi: 10.1038/s41467-021-27569-x (PMC8664878; doi:10.1038/s41467-021-27569-x)
Supplement: Supplementary file 3 — Description of Additional Supplementary Files [file 41467_2021_27569_MOESM3_ESM.pdf]

Description of Additional Supplementary Files

File Name: Supplementary Data 1
Description: The Dataset 1 includes database generated during the study.
