# Supplementary material for: Stable isotopes in global lakes integrate catchment and climatic controls on evaporation
Source: Nat Commun. 2021 Dec 10;12:7224. doi: 10.1038/s41467-021-27569-x (PMC8664878; doi:10.1038/s41467-021-27569-x)
Supplement: Supplementary file 2 — Peer Review File [file 41467_2021_27569_MOESM2_ESM.pdf]

nature portfolio

Peer Review File

Stable isotopes in global lakes integrate catchment and climatic controls on evaporation

Open Access This file is licensed under a Creative Commons Attribution 4.0 International License, which permits use, sharing, adaptation, distribution and reproduction in any medium or format, as long as you give appropriate credit to the original author(s) and the source, provide a link to the Creative Commons license, and indicate if changes were made. In the cases where the authors are anonymous, such as is the case for the reports of anonymous peer reviewers, author attribution should be to 'Anonymous Referee' followed by a clear attribution to the source work. The images or other third party material in this file are included in the article's Creative Commons license, unless indicated otherwise in a credit line to the material. If material is not included in the article's Creative Commons license and your intended use is not permitted by statutory regulation or exceeds the permitted use, you will need to obtain permission directly from the copyright holder. To view a copy of this license, visit http://creativecommons.org/licenses/by/4.0/.

Reviewers' Comments:

Reviewer #1:
Remarks to the Author:
Comments on the manuscript “Stable isotopes in global lakes integrate catchment and climatic controls on evaporation” submitted to NG first and transferred to Nature Communications.
This study analysed the stable isotope composition of 1,257 global lakes to explore the effects of catchment characteristics and climatic variables on lake evaporation losses. This is an interesting study. The manuscript is overall well-written, but I have following concerns/comments on the current manuscript.

Major comments
1. The reliability of estimated lake evaporation losses is unclear, compared to estimates based on other methods (e.g. energy balance, combination equations). Many studies on lake evaporation losses are existing, the MODIS global reservoir/lake evaporation product is also just released https://modis-land.gsfc.nasa.gov/pdf/MOD28_ATBD_Jan2021.pdf. I believe most readers would expect a comparison of the estimated lake evaporation losses in this study and other existing studies or data, in order to understand the reliability of the current study. Therefore, I advise the authors to add a thorough discussion of the reliability of their estimates by putting them in context of existing studies.

2. Two very important variables are not considered in the analysis, that is, lake surface temperature (LST) and lake depth.

About LST: the authors stated (on Line 264 of the manuscript) the lake surface temperature as one of key parameters, but it is a pity that they did not consider LST (rather only air temperature) in their analysis and the Random-Forest based modelling. Many LST datasets are being made available, e.g. https://land.copernicus.eu/global/products/lswt and http://www.laketemp.net/home/, I strongly advise the authors consider LST in their analysis.

About lake depth: the authors mentioned the reason why the lake depth was not used on Line 147 of the Supplementary Information. However, lake depth is indeed an important parameter affecting the lake evaporation process particularly the lake heat storage (e.g. Duan and Bastiaanssen, 2015; Finch and Calver, 2008; Zhao et al. 2020). Actually there are lots of data sources providing lake depth, which is worthwhile for exploring. For example, HydroLAKES, Global Lakes and Wetlands Database, and the Global Lake Database (GLDB, Toptunova et al., 2019) used in ERA reanalysis product for global lakes.

References mentioned:
Duan, Z., & Bastiaanssen, W. G. M. (2015). A new empirical procedure for estimating intra-annual heat storage changes in lakes and reservoirs: review and analysis of 22 lakes. Remote Sensing of Environment, 156, 143-156.

Finch, J., & Calver, A. (2008). Methods for the quantification of evaporation from lakes. http://nora.nerc.ac.uk/14359/1/wmoevap_271008.pdf

Toptunova, O., Choulga, M., & Kurzeneva, E. (2019). Status and progress in global lake database developments. Advances in Science and Research, 16, 57-61

Zhao, G., Gao, H., & Cai, X. (2020). Estimating lake temperature profile and evaporation losses by leveraging MODIS LST data. Remote Sensing of Environment, 251, 112104.

3. Lack of essential details (e.g. time period, temporal scales) on dataset and processing. Many different variables were used in this study, however, lots of necessary details on the data are missing in the current manuscript.

For example, what are the considered time period for the variables? On what temporal scales? Are all variables available on the same time period and temporal scales to match up for the further analysis?

Isotopic mass balance requires total water inflow into the lake, and the lake outflow including surface and subsurface flows, how the authors determine these variables and based on what data sources? (seems no description was given in the manuscript).

4. Data availability. The author provided the link https://nucleus.iaea.org/sites/ihn , but I cannot find the global lakes dataset there. Please check and ensure the dataset are accessible for the readers.

Other specific comments
Figure 1. It might be better to explain Number of data 7457 in the caption as well.

Line159-161. Please specify "evaporation losses", the evaporation rate, or total evaporation losses (rate * lake area)

Line 227-228. Bowen ratio seems inappropriate for being the variable, try to link the physical variables (e.g. relative humidity and air temperature) to align with the mentioned wind speed and solar radiation.

Line 238-239. Specify that generally deep lakes where have considerable changes in lake heat storage, more details on this can be found in publications e.g. (Duan and Bastiaanssen, 2015; Finch and Calver, 2008; Zhao et al. 2020).

Reviewer #2:
Remarks to the Author:
General remarks
The manuscript describes the results of an advanced statistical analysis of a global lake water isotope data set. It aimed at identifying key drivers in evaporation from lakes and analyzed various parameters and (briefly) compared their isotope-model results with non-isotope models. The authors further identified differences to theoretical predictions from e.g. the popular C-G model and demonstrated that energy parameters might play a more important role than previously thought.

Technically, structure, language, length and number of figures of manuscript are fine. Literature is appropriate and up-to-date. Methods are well explained and, where necessary, the online methods section provides full details.

The study is interesting to a broader scientific community as it uses a rather specialised methodology (stable isotope hydrology) but findings and conclusions are explained in a way that also neighbouring field of science (ecology, water management, climatology etc) can benefit from this.

Overall, I fully support the publication of this interesting global assessment of stable isotope data in n-Comms. The study is definitely one of the few studies that successfully make a step forward in the interpretation of water stable isotope data with respect to ongoing climate change and identifying key parameters that will change in future. While evaporative model approaches are often restricted to the catchment scale, this is a good example how isotope data can be used to get a better understanding of the controlling factors in water balance also on a larger, global scale.

Specific comments
L92 How was the seasonal variation of a single lake treated in this approach? Depending on lake size, the isotope data will change from the cool to warm season, following the local evaporation line, at

least in temperate climates. In such a case, how was d18O_L (L277) calculated? From the methods, I understand that in such a case the median value over all available data points from a single lake was calculated ("median per lake"). This should be mentioned as this obviously different from the median isotope values discussed in this section (and Figure 1), which refer to a "median of values from lakes of a climate type".

Further: Are the d18O values discussed in L92 ff. and Figure 1 isotope values (d18O_L and d2H_L) on the VSMOW-SLAP scale or somehow normalized values (capDeltadelta; Δδ)? This is unclear. I assume that original isotope values are presented, but in L86 it was just stated that "Each lake's isotopic composition was normalized to the catchment-weighted precipitation input". This can be confusing to the reader. Therefore, by using subscripts such as L or P, please use them consequently throughout the entire publication.

L96 To what does these two delta-values refer to? Global averages? Please specify.

L103 LEL was not yet defined.

L108 It should be briefly mentioned that d18O values discussed here (e.g. -5.5 ‰) are normalized isotope values. I know, this was mentioned before but to avoid confusion, I think this might be necessary. For example change to "…the lake water Local Evaporation Line (LEL) intersection of normalized isotope values was slightly lower for d18O …."

Figure 2 caption Please mention that values shown for intersections refer to oxygen isotopes only.

L128 Perhaps better write "LEL slopes"?

Figure 4 Please indicate in figure or caption in which axis direction a variable is more important i.e. explains more of the evaporative loss in the model.

L189 See before. How can this be inferred from the position of the points shown in Fig. 4? I suspect not every reader is familiar with this plot.

Supplementary material appears fine.

Technical comments
L50 space character missing 'surface-'
L53 etc -> might change to "among others" or "and other" (or skip)
L95 delta symbol not italic; please check throughout the manuscript (e.g. L110, L114, L128). Be consistent, either italic or not.
L125 Why quotation marks for LEL but not for GMWL?
L279 number equation

Reviewer #3:
Remarks to the Author:
The authors came up with the most comprehensive water isotope data base on lakes accross the globe. This is unprecedented data. The authors employed the well proven isotope balance formulation- the Craig Gordon evaporation mdoel- to compute the evaporative water loss of lakes with respect to their inflows- i.e. E/I ratio. They authors then compare lakes accross the various geo/climatic regions based on isotope behaviors to identify key environmental factos that affect/control the isotope composition of the lakes.

The manuscript merits publication in Nature Communications. However, there needs to be

clarifications regards a few questions raised in the annotated PDF manuscript. The most important questions to be addressed are the following

1. The computation that globally the E/I is equal to 0.2 looks a signifcant underestimation of evaporative water loss in catchments. This finding must be clarified. If this value represent the evaporative water loss with respect to inflows in lakes the value may be agreeable. However, this ratio underestimates the role of evaporative water loss in catchments if whole catchment is considered. The authors must clarify what the 0.2 fraction stands for.

2. ON the data selection approaches: The authors argue that datasets which show discripancy in E/I computation when O and H were used have been eliminated from the dataset. However, as widely published and also aurged by the authors (line 136-140) there discripancy between O and H method a well known problem. What percent of the dataset has been eliminated/not considered in the analysis must be reflected in the manuscript.

3. ON comparing the 18O-2H at the Intersection of LEL and GMWL with the mean 18O-2H of Local Preciption: The general finding that there is a difference between the two is a signficant finding! However for at least one case the reasoning behind such departure needs to be elaborated further or explined. The case is when the intersection point shows enrichment compared to the precpitation. The authors argue that is because of pre-evaporation within the catchment that makes intersection points to show higher values. However, as shown in Gonfiantini 1986 and Kebede et al., 2009; pre-evaporation of the inflowing water to the lakes would not alter the slope of the LEL. Evaporation within lakes only pushes the isotopic composition along the LEL without altering the slope. Therefore the authors must find alternative explanation for the descripancy between the two values.

3. On comparing isotope based E/I with environmental variables: The manuscript depicts the various environmetnal and climatic factors as controls on the isotopic compositon. However, in my view some of these parameters are causaly related to the E/I and others are just correlational. See the attached annotated PDF for details.

REVIEWER COMMENTS

We would like to acknowledge the reviewers for the general positive opinion and support of our work, the through revision and ideas. We provided the detailed answer on each comment below.

Dr Yuliya Vystavna on behalf of all authors

REVIEWER 1 (REMARKS TO THE AUTHOR):

Comments on the manuscript "Stable isotopes in global lakes integrate catchment and climatic controls on evaporation" submitted to NG first and transferred to Nature Communications. This study analysed the stable isotope composition of 1,257 global lakes to explore the effects of catchment characteristics and climatic variables on lake evaporation losses. This is an interesting study. The manuscript is overall well-written, but I have following concerns/comments on the current manuscript.

We would like to thank Reviewer 1 for the positive opinion about our work, for important comments and for the time spent on the paper reading and reviewing. We answered all concerns/comments and provided the detailed explanations below.

Major comments

Comment 1: The reliability of estimated lake evaporation losses is unclear, compared to estimates based on other methods (e.g. energy balance, combination equations). Many studies on lake evaporation losses are existing, the MODIS global reservoir/lake evaporation product is also just released *https://modis-land.gsfc.nasa.gov/pdf/MOD28_ATBD_Jan2021.pdf.* I believe most readers would expect a comparison of the estimated lake evaporation losses in this study and other existing studies or data, in order to understand the reliability of the current study. Therefore, I advise the authors to add a thorough discussion of the reliability of their estimates by putting them in context of existing studies.

Reply 1: In our study we used the classical and well proven isotope mass balance method to estimate evaporation losses of the lake in relation to the lake inflow (E/I ratio) *(Rozanski et al. 2001, Gibson et al. 2002, 2014, 2021, Jasechko et al. 2013*). The reliability of the method has been validated in the technical documentation (*Rozanski et al. 2001*) and multiple peer-reviewed papers (i.e. *Craig and Gordon 1965, Gibson et al. 1993, 2002, 2014, 2021, Vallet-Colomb et al. 2008, Kebede et al. 2009, Jasechko et al. 2013, 2014*). Compared to other methods mentioned in the paper (**L. 54-55**) and highlighted by the Reviewer, isotope mass balanced method allows to assess the ability of the lake-catchment system to compensate the evaporation losses of the lake by its inflow (*Rozanski et al. 2001, Gibson et al. 2002, 2014, 2021*) (**L. 56-59**). Our findings provide a reasonable first-order proxy of the evaporation losses in lakes in relation to the inflow at the global scale, but also describe hydrometeorological and catchment factors that can impact lake evaporation status and can be used for global, regional, and even local assessments and paleoclimate interpretation. Therefore, we are not considering the lake as an evaporation pan but looking at the catchment scale (**L. 74-75**).

We agree with the reviewer's view on the potential to couple remote sensing with stable water isotopes. As compared to the satellite observations driven approach, the physical environmental tracers, namely isotopes, trace naturally occurring variations arising from phase changing and mixing as water passes through the hydrological cycle integrating the in-situ hydroclimate conditions (*Craig and Gordon 1965, Gibson et al. 1993, Jasechko et al. 2013, 2014*). Particularly, being a natural hydrological tracer, stable water isotopes in lakes can contribute to

verify satellite-based approaches with complementary data about evaporation. We are also mentioning this explicitly in the text of the revised version (**L. 267-273**):

"***Compared to remote sensing-based assessments of changes in global lakes, stable isotope variations directly reflect all of the phase changes and mixing that occurs as water passes through the entire catchment-lake hydrological cycle, thereby integrating regional and local in-situ hydroclimate conditions and distinctions that may not be evident from satellite observations[15,19]. Accordingly, as a natural hydrological tracer, long-term stable water isotope monitoring of Earth's lakes complements and contributes to validation and fine tuning of satellite-based approaches and by providing explicit data about their evaporation status***."

Our study is a first step towards this goal. We also used extensive manual adjustments that should minimize the reference data error and modelling errors often deriving with the remote data processing (*Messager et al. 2016*).

As described in the provided pdf file with the link on *Zhao et al*. Collection 6.1 MODIS Global Reservoir Product Algorithm Theoretical Basis Document (ATBD) Version 1.0 MODIS is a product to approximate evaporation in artificial lakes (water reservoirs). ***The uniqueness of our database lies in the lab-measured isotope data from more than 1,000 lakes including small to great lakes, natural and artificial lakes, with sufficient global geographical coverage.*** Compared to artificial lakes, natural lake systems are sufficiently 'old' to represent the long-term environmental conditions and can have a longer response on the hydroclimate variations that are generally harder to evaluate. While we agree that the research on artificial lakes is important, as these are constructed more and more all around the world, changing the hydrological cycle and are a powerful sign how humanity change the earth's surface, we would also like to point out that environmental parameters are much easier available for reservoirs/artificial lakes as they are constructed with known properties (inflow and outflow, size, depth, etc) and often continuously monitored (air and water temperature, relative humidity, etc). In contrast, our global lake data base shows a large variety in terms of size, known parameters, remoteness etc. This makes our database more exclusive and less biased towards artificial lakes. Our database fills a significant knowledge gap regarding natural lakes, where global information is both scarce and inconsistent thereby complicating the environmental, social and economic assessments (*Messager et al. 2016*).

Comment 2: Two very important variables are not considered in the analysis, that is, lake surface temperature (LST) and lake depth.

Reply 2: LST and lake depth application is discussed below (Comments 3 and 4).

Comment 3: About LST: the authors stated (on Line 264 of the manuscript) the lake surface temperature as one of key parameters, but it is a pity that they did not consider LST (rather only air temperature) in their analysis and the Random-Forest based modelling. Many LST datasets are being made available, e.g. *https://land.copernicus.eu/global/products/lswt* and *http://www.laketemp.net/home/*, I strongly advise the authors consider LST in their analysis.

Reply 3: We agree with the reviewer that additional parameters can be considered. However, classical isotope-mass balance method based on the assumption that the mean annual temperature of the lake (LST) is identical to the mean annual surface air temperature in the area (*Rozanski et al. 2001, Gibson et al. 2002, 2014, 2021, Jasechko et al. 2013*). While the LST can be different from the air, it is likely biased at the annual scale that is considered in this study.

Following the Reviewers advice, we retrieved the LST data from the databases proposed by the Reviewer. In total, we had LST data only for 1/3 of the lakes mainly largest by surface area, thereby introducing a bias. Additionally, the correlation between retrieved LST and air temperature was very high (R^2=0.61) and confirms the reliability of the undertaken assumption. The explanation was added in the method section in the Supplementary Information (p.13):

"Lake surface temperature (LST) was not included in the RF because we were able to retrieve LST data only for ca. 30% of lakes from our database and these were strongly biased to large lakes. Also, classical isotope-mass balance modelling approach based on the assumption that the mean annual temperature of the lake is identical to the mean annual surface air temperature in the area[10,27,28]. The correlation between retrieved lake surface temperature and air temperature was high (R^2=0.61) that confirm the reliability of the undertaken assumption. While the temperature of lake can be different from the air, it is likely biased at the annual average scale that is considered in this study".

Comment 4: About lake depth: the authors mentioned the reason why the lake depth was not used on Line 147 of the Supplementary Information. However, lake depth is indeed an important parameter affecting the lake evaporation process particularly the lake heat storage (e.g. Duan and Bastiaanssen, 2015; Finch and Calver, 2008; Zhao et al. 2020). Actually there are lots of data sources providing lake depth, which is worthwhile for exploring. For example, HydroLAKES, Global Lakes and Wetlands Database, and the Global Lake Database (GLDB, Toptunova et al., 2019) used in ERA reanalysis product for global lakes.

References mentioned:

Duan, Z., & Bastiaanssen, W. G. M. (2015). A new empirical procedure for estimating intra-annual heat storage changes in lakes and reservoirs: review and analysis of 22 lakes. Remote Sensing of Environment, 156, 143-156.

Finch, J., & Calver, A. (2008). Methods for the quantification of evaporation from lakes. http://nora.nerc.ac.uk/14359/1/wmoevap_271008.pdf

Toptunova, O., Choulga, M., & Kurzeneva, E. (2019). Status and progress in global lake database developments. Advances in Science and Research, 16, 57-61

Zhao, G., Gao, H., & Cai, X. (2020). Estimating lake temperature profile and evaporation losses by leveraging MODIS LST data. Remote Sensing of Environment, 251, 112104.

Reply 4: We are very thankful for the suggested variables, papers and databases. These are great resources and points to be considered.

Heat storage: The approach proposed by *Zhao et al. (2020)* is important to understand the lake/reservoir seasonality and its dynamic under the climate change. We see a great potential here to couple this approach in the future with stable water isotopes. Here, a global network of stable water isotopes in lakes can contribute to verify satellite-based approaches with complementary data about evaporation. Our study shows that isotopes in fact, have this potential. The MODIS approach was tested on 11 lakes and revealed that for the shallow lakes heat storage can be neglected, but for deep lakes (>10 m), heat storage changes can be equivalent to more than 50% of the net radiation and lead to a significant shift in evaporation seasonality (*Zhao et al. 2020*). Additionally, *Zhao et al. (2020)*, but also other authors (*Duan and Bastiaanssen 2015* with an analysis of 22 lakes) stated that the heat storage is a *seasonal* phenomenon, and the errors tend to be cancelled out in the annual average values. As evaporation seasonality related to intra-annual variations were not considered in

our study (we used annual averaged data that integrates the seasonal variations), we didn't integrate heat storage in the study, but included other parameters related to the heat storage such as the Bowen ratio, latent and sensible heat fluxes. However, as mentioned above - we fully agree with Reviewer that this parameter is important to be considered to study seasonal evaporation shifts and added the reference of *Zhao et al. (2020)* in the manuscript (**L. 53**).

Lake depth: We agree with Reviewer that satellite based remote sensing is a useful tool to allow effective monitoring of lakes and particularly, for the shallow lakes (*Pope et al. 2016, Sneed and Hamilton 2011, Kourzeneva et al. 2012, Purchla et al. 2018*). However, for the lakes deeper than 1.5 m and lakes with significant fluctuation of water depth (i.e., coastal lakes), remote sensing can provide unrealistic data due to the following constraints (*Pope et al. 2016, Sneed and Hamilton 2011, Kourzeneva et al. 2012, Purchla et al. 2018, Messager et al. 2016*):

1. Difficulty to measure the true reflectance of dark/deep lakes.
2. Assumption about minimal quantities of suspended and dissolved solids in lake water.
3. The requirement for the smooth (not wind roughened) lake surface.
4. Homogenous and low slope lake bottoms.
5. With regards to the HydroLakes approach: assumptions of certain type of lake formation, where surrounding topography determines lake depth. However, in some cases of e.g., glacial lakes of high latitudes, solution lakes, oxbow and permafrost lakes this will not necessarily result in accurate depth estimations.

When compared with bathymetry and other *in-situ* observations, the retrieved data from the global lake database and HydroLakes depth data were significantly different for many lakes. Therefore, we kept only the data that were comparable between these two methods, avoiding the over/under estimation of the depth importance. In total, we had 538 lakes with the lake depth (average). Furthermore, we need to consider that the average lake depth is not always a correct indicator as the heat can be stored at the deeper part of lakes (*Zhao et al. 2020, Duan and Bastiaanssen 2015*) adding an additional uncertainty to the calculations. When adding obtained data on average depth to the random forest model, we have found that for tropic, arid and polar lakes, the depth parameter had very low Mean Decrease Gini (IncNodePurity) that indicated the data heterogeneity in tree splits and low classification performance. Another problem was associated with the low importance of the depth parameter (IncMSE is < 10-20%) in all climate types. In tropical, arid, continental and polar lakes the random forest model performance was decreased (~30%) after adding the average depth. But for lakes in temperate climate, the random forest model performance increased on 15%, however the importance of the average depth remained very low. We suggest that by adding the depth, random model performance and explanation for the evaporation losses can be improved, but more accurate depth data in global lakes and additional analysis on the selection of the variable (averages vs maximum) are needed.

Comment 5: Lack of essential details (e.g., time period, temporal scales) on dataset and processing. Many different variables were used in this study, however, lots of necessary details on the data are missing in the current manuscript. For example, what are the considered time period for the variables? On what temporal scales? Are all variables available on the same time period and temporal scales to match up for the further analysis?

Reply 5: Years are included in the database and will be available online after the paper acceptance. At the temporal scale, the database covers a period from 1956 to 2020, where most of lakes (64% of total lakes) were sampled at the period from 1990 to 2010.

The access to well distributed temporal hydrological data derived from the in-situ measurements (but also from the satellite observation) is a complex task (*Sivapalan and Bloschl 2015, Evaristo and McDonell 2017*). Citing *Sivapalan and Bloschl (2015)* and refering to *Evaristo and McDonell (2017)*, we would like to state that to obtain 30-year records of precipitation and runoff data at one location, would be the same as the sensitivity obtained from a spatial transect of precipitation and runoff at the same time. In other words, the short record contains the same dynamics as the legacy of processes apparent in the spatial differences. However, if a long-term (slow) component is also present, as lakes water residence time, this is no longer the case, as the 30-year temporal record will only sample the fast processes, while the spatial transect will show the legacy effects of both the fast and slow processes.

In our study, data for most of lakes were only for one year and only 15 lakes were sampled for 5-7 years. Therefore, due to low amount of time series data, we were not able to construct significant time trends. For these lakes with 5-7 years data, time series trends of $\delta^{18}O$ were not significant or at low significance likely indicating that natural surface water systems, such as lakes, typically function close to their hydrological and isotopic steady state attained in the course of their long history (*Isokangas et al. 2015*). This is consistent with *Gibson and Reid (2014)*, who showed on 20 years data of the isotope in lakes that the year-to-year variations in precipitation on the lake are not very influential in determining the interannual variations and this variability is low (ca. 10%) (*Rozanski et al. 2001*). *Gibson et al. (2021)* has tested the reproducibility of the results for specific watersheds in Canada, with data obtained from earlier decades (1990 and 2000) and found these agreed with recent estimates to with 7 % and 3 % respectively, despite being based on different periods of record for climate, discharge, and isotopic composition of streamflow.

Longer observation (up to 100 years and longer) period are needed to determine the isotope and water balance time series, particularly, considering that moderate and large size lakes have residence time > 30 years and even > 100 years. Lakes drain catchment hydrological reservoirs of different age and linking hydrological and climate parameters to particular year is not representative. Obtained remote sensing data on residence time were available only for less than 10% of lakes (*HydroLakes and Messager et al. 2016*) and these data were in the discrepancy with other published scientific data (can be linked to estimation method) and were not reliable for the modelling. From the other side, remote sensing data are available mainly since 2002. Therefore, we took catchment weighted hydrological and climate parameters averaged during the maximum available range (2002-2020) and assume that the isotopic composition of the lake integrates long term period (*Jasechko et al. 2013,2014, Gibson and Reid 2014*).

Like previous studies, we suggest that long term (30 or more years, at least at annual resolution) observation of isotopes and collecting of remote sensing data on hydrological, meteorological and other parameters will improve the interpretation (*Jasechko et al. 2013, Gibson et al. 2021*). However, in this study, we focused on the average state of the lake system that were characterised by long-term (20-30 years) means of the environmental parameters that is a typically of interest for hydrological practices as lakes systems are sufficiently 'old' (*Rozanski et al. 2001*). A statement about the data time frame is added in the method section of the Supplementary information (p.7):

"The database covers the period 1956 to 2020, where most lakes (64%) were sampled sporadically from 1990 to 2020. Only 15 out of 1,257 lakes were sampled systematically for 5-7 years on average, which did not allow us to investigate the time trends. Like in other studies[4-6], we assumed that lakes operate close to their hydrological and isotopic steady states attained during their long history[7] with low interannual variation in the time frame covered in the study".

Comment 6: Isotopic mass balance requires total water inflow into the lake, and the lake outflow including surface and subsurface flows, how the authors determine these variables and based on what data sources? (seems no description was given in the manuscript).

Reply 6: Use of isotope-mass balance calculations to evaluate vapour outflow from a surface water body essentially reduces to solution of a two-component mixing problem. This involves balancing the volume of lost vapour with outflowing lake water to yield input water. The common assumption is done for the hydrologic steady state conditions (i.e., undergoing evaporation while maintaining constant volume). This assumption was applied for the isotope-mass balanced calculations like in other studies (*Gibson et al. 1993, Jasechko et al. 2013, Kebede et al. 2009*). The steady-state conditions characteristics of lakes are defined by local climate, morphological settings, and prevailing hydrological regime (*Isokangas et al. 2015*).

The detailed description of the method is mentioned in the text (**L. 56-59, 288-290**) and provided in the method section of the Supplementary Information (p. 14-15)

Comment 7: Data availability. The author provided the link *https://nucleus.iaea.org/sites/ihn* , but I cannot find the global lakes dataset there. Please check and ensure the dataset are accessible for the readers.

Reply 7: The database will be available after the paper acceptance as it is considered in the journal policy.

Other specific comments

Comment 8: Figure 1. It might be better to explain Number of data 7457 in the caption as well.

Reply 8: The Figure 1 caption was corrected, and data points were added in the revised version of the manuscript:

"Figure 1. Distribution of $\delta^{18}O_L$ composition (n=7,457 data points) in 1,257 lakes around the globe and in climatic zones based on the Köppen-Geiger climate classification[16,17]. Median and ranges of $\delta^{18}O_L$ for all lakes by climatic zone are depicted in box-and-whiskers plots. The climate map was generated according to Kottek et al. (2006)[17]".

Comment 9: Line159-161. Please specify "evaporation losses", the evaporation rate, or total evaporation losses (rate * lake area)

Reply 9: The classical isotope mass balance method has been used to estimate fluxes regulating the water balance of lakes, including evaporation (E) to inflow (I) ratios (E/I) (i*.e., Gonfiantini 1986, Rozanski et al. 2001, Gibson et al. 1993, 2002, 2021, Kebede et al. 2009*). Therefore, we refer to the evaporation losses relative to water inflow in the lake. The detailed description of the method is mentioned in the text (**L. 56-59, 288-290**) and provided in the method section of the Supplementary Information (**p. 14-15**).

Comment 10: Line 227-228. Bowen ratio seems inappropriate for being the variable, try to link the physical variables (e.g. relative humidity and air temperature) to align with the mentioned wind speed and solar radiation.

Reply 10: Applying machine learning algorithm (Figure 4), we have found that Bowen ratio was important for tropical lakes. However, we agree with the Reviewer that Bowen ratio was less

important variable than relative humidity, particularly in arid lakes, but also small and moderate size lakes. The statement was corrected as follows:

L.227-229: "*…together with driving forces of water evaporation (air temperature, relative humidity, wind speed and solar radiation)".*

Comment 11: Line 238-239. Specify that generally deep lakes where have considerable changes in lake heat storage, more details on this can be found in publications e.g. (Duan and Bastiaanssen, 2015; Finch and Calver, 2008; Zhao et al. 2020).

Reply 11: Our target is to estimate and explain the evaporation in relation to inflow rate (E/I) that is a measure of the ability of the lake to compensate the evaporation losses (i.e., *Gonfiantini 1986, Rozanski et al. 2001, Gibson et al. 1993, 2002, 2021, Kebede et al. 2009*). For this purpose, we used the annual means that characterise the average state of the lake systems. As mentioned above, deep lakes (>10 m), heat storage changes can be equivalent to more than 50% of the net radiation and lead to a significant shift of evaporation seasonality Zhao et al. (2020). But we used annual average data and refer to *Zhao et al. (2020)* in **L. 53** concerning the heat storage (details are described in Comment 4).

REVIEWER 2 (REMARKS TO THE AUTHOR):

General remarks

The manuscript describes the results of an advanced statistical analysis of a global lake water isotope data set. It aimed at identifying key drivers in evaporation from lakes and analyzed various parameters and (briefly) compared their isotope-model results with non-isotope models. The authors further identified differences to theoretical predictions from e.g. the popular C-G model and demonstrated that energy parameters might play a more important role than previously thought. Technically, structure, language, length and number of figures of manuscript are fine. Literature is appropriate and up-to-date. Methods are well explained and, where necessary, the online methods section provides full details.

The study is interesting to a broader scientific community as it uses a rather specialised methodology (stable isotope hydrology) but findings and conclusions are explained in a way that also neighbouring field of science (ecology, water management, climatology etc) can benefit from this. Overall, I fully support the publication of this interesting global assessment of stable isotope data in n-Comms. The study is definitely one of the few studies that successfully make a step forward in the interpretation of water stable isotope data with respect to ongoing climate change and identifying key parameters that will change in future. While evaporative model approaches are often restricted to the catchment scale, this is a good example how isotope data can be used to get a better understanding of the controlling factors in water balance also on a larger, global scale.

We would like to thank Reviewer 2 for the overall positive opinion and support of our work, for important comments and for the time spent on the paper reading and reviewing. We answered all concerns/comments and provided the detailed explanations below.

Specific comments

Comment 12: L92 How was the seasonal variation of a single lake treated in this approach? Depending on lake size, the isotope data will change from the cool to warm season, following the local evaporation line, at least in temperate climates. In such a case, how was $d18O_L$ (L277) calculated?

Reply 12: We agree with the Reviewer that the seasonal aspect is important in the isotope mass balance study. All lake systems undergo some short-term fluctuation caused by the seasonality of individual hydrological components (precipitation, inflow, lake water) (i.e., *Rozanski et al. 2001*). The isotopic seasonality was considered in our study based on the following assumptions and calculations: (i) groundwater that is often the dominant inflow component in the lake was assumed to have no seasonal variations. The assumption is based on previous studies that showed that groundwater has water transit time more than 1 year and biases the seasonality of precipitation (i.e. *Jasechko et al. 2019*), (ii) precipitation isotopic seasonality was removed by the normalization of the isotopic signation of the lake by the catchment weighted precipitation that refer to the 'evaporative enrichment' ($\Delta_{L\text{-}P}\delta^{18}O$), (iii) according to the sampling method (i.e*. Rozanski et al. 2001*), the sampling on the isotope analysis should be done during the mixing period or the lake water profile should be sampled and converted to the average that represents the mixing conditions or alternatively, lake outflow should be sampled. Therefore, we assumed that the sampling was done according to the guidelines and the isotopic stratification of the lake column was integrated in the lake isotopic fingerprint. It is also assumed that lakes were sampled during the evaporation period that is the main task of the isotope mass balance studies (*Rozanski et al. 2001, Gibson et al. 1993, 2002, 2021*).

The statement was added in the method section of the Supplementary information (**p. 12**):

"Seasonal effect was eliminated by the conversion of raw isotope values in lake median value and so season was not considered in the model. Additionally, considering that all lake systems undergo short term fluctuation caused by the seasonality of individual hydrological components (precipitation, inflow, lake water)[27]. Isotopic seasonality was considered in our study based on the following assumptions and calculations: (i) groundwater that is often the dominant inflow component in the lake was assumed to have no seasonal variations. The assumption is based on previous studies that showed that groundwater has water transit time more than 1 year and biases the seasonality of precipitation[28], (ii) precipitation isotopic seasonality was removed by the normalization of the isotopic signation of the lake by the catchment weighted precipitation that refer to the 'evaporative enrichment' ($\Delta_{L\text{-}P}\delta^{18}O$), (iii) according to the sampling method[27], sampling for isotope analysis should be done during the mixing period or the lake water profile should be sampled and converted to the average that represents the mixing conditions or alternatively, lake outflow should be sampled. Therefore, we assumed sampling was done according to these guidelines and the isotopic stratification of the lake column was integrated in the lake isotopic fingerprint. It is also assumed that lakes were sampled during the evaporation period which is the reason for conducting isotope mass balance studies [10,11,27]."

Comment 13: From the methods, I understand that in such a case the median value over all available data points from a single lake was calculated ("median per lake"). This should be mentioned as this obviously different from the median isotope values discussed in this section (and Figure 1), which refer to a "median of values from lakes of a climate type".

Reply 13: The caption of Figure 1 was changed accordingly. The statements about the median values were also revised in the manuscript and in the Supplementary information (p. 8).

"Figure 1. Distribution of $\delta^{18}O_L$ composition (n=7,457 data points) in 1,257 lakes around the globe and in climatic zones based on the Köppen-Geiger climate classification[16,17]. Median and ranges of $\delta^{18}O_L$ for all lakes by climatic zone are depicted in box-and-whiskers plots. The climate map was generated according to Kottek et al. (2006)[17]".

Comment 14: Further: Are the d18O values discussed in L92 ff. and Figure 1 isotope values (d18O$_L$ and d2H$_L$) on the VSMOW-SLAP scale or somehow normalized values (capDeltadelta; Δδ)? This is unclear. I assume that original isotope values are presented, but in L86 it was just stated that "Each lake's isotopic composition was normalized to the catchment-weighted precipitation input". This can be confusing to the reader. Therefore, by using subscripts such as L or P, please use them consequently throughout the entire publication.

Reply 14: Thank you for spotting it. We agree with the reviewer. The text was modified accordingly so that it is clear when raw ($\delta^{18}O_L$ and $\delta^{18}O_P$) or normalized ($\Delta_{L-P}\delta^{18}O$) values are used.

Comment 15: L96 To what does these two delta-values refer to? Global averages? Please specify.

Reply 15: We agree with the reviewer. The text was modified accordingly so that it is clear when raw ($\delta^{18}O_L$ and $\delta^{18}O_P$) or normalized ($\Delta_{L-P}\delta^{18}O$) values are used.

Comment 16: L103 LEL was not yet defined.

Reply 16: LEL was defined in the Introduction (**L. 61**) as ***'local evaporation line'***.

Comment 17: L108 It should be briefly mentioned that d18O values discussed here (e.g. -5.5 ‰) are normalized isotope values. I know, this was mentioned before but to avoid confusion, I think this might be necessary. For example change to "…the lake water Local Evaporation Line (LEL) intersection of normalized isotope values was slightly lower for d18O …."

Reply 17: corrected as proposed (Comments 15 and 16).

Comment 18: Figure 2 caption Please mention that values shown for intersections refer to oxygen isotopes only.

Reply 18: The figure caption was corrected as suggested:

"Figure 2. The LEL lines based on the isotopic values of lakes normalized to their weighted catchment precipitation and intersection of these lines with the global meteoric water line (GMWL). Intersection values refer to $\delta^{18}O$ values."

Comment 19: L128 Perhaps better write "LEL slopes"?

Reply 19: corrected as proposed (**L. 129**).

Comment 20: Figure 4 Please indicate in figure or caption in which axis direction a variable is more important i.e. explains more of the evaporative loss in the model.

Reply 20: Arrows to indicate the direction for the variable importance was added in Figure 4.

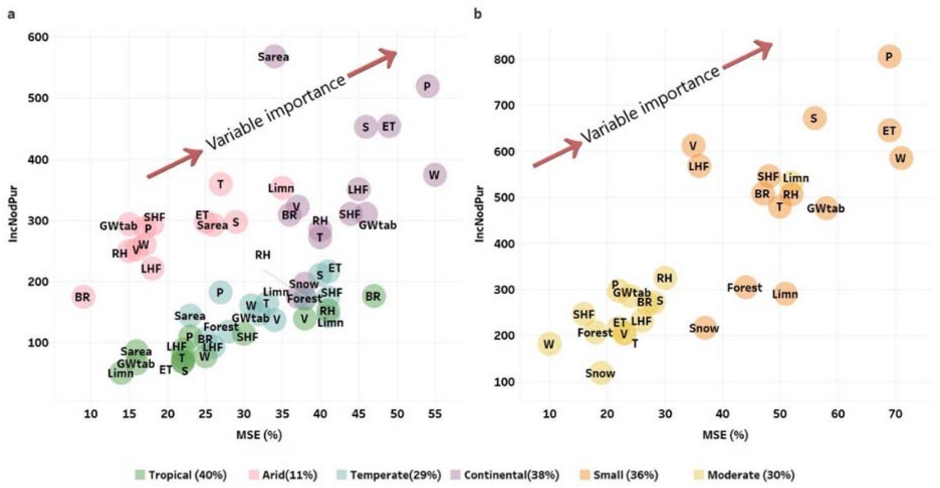

L. 188: "Variable importance is indicated by the arrow direction."

Comment 21: L189 See before. How can this be inferred from the position of the points shown in Fig. 4? I suspect not every reader is familiar with this plot.

Reply 21: The arrows indicating the direction for the variable importance was added in Figure 4 (details in Comment 20).

Supplementary material appears fine.

Technical comments

Comment 22: L50 space character missing 'surface-'

Reply 22: Corrected.

Comment 23: L53 etc -> might change to "among others" or "and other" (or skip)

Reply 23: Corrected.

Comment 24: L95 delta symbol not italic; please check throughout the manuscript (e.g. L110, L114, L128). Be consistent, either italic or not.

Reply 24: Corrected and checked.

Comment 25: L125 Why quotation marks for LEL but not for GMWL?

Reply 25: The figure 2 and the caption was corrected:

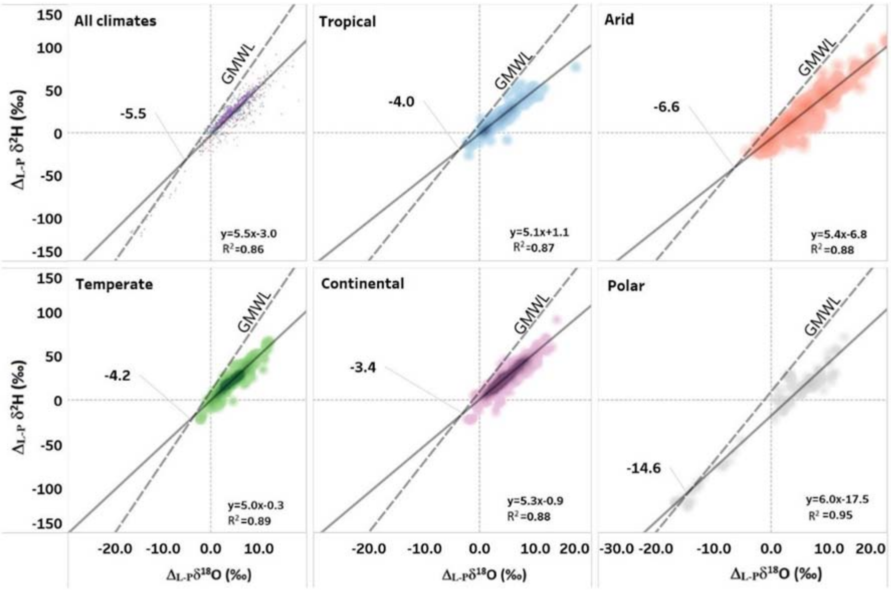

“Figure 2. The LEL lines based on the isotopic values of lakes normalized to their weighted catchment precipitation and intersection of these lines with the global meteoric water line (GMWL). Intersection values refer to δ^{18}O values.”

Comment 26: L279 number equation

Reply 26: Corrected.

REVIEWER 3 (REMARKS TO THE AUTHOR):

The authors came up with the most comprehensive water isotope data base on lakes accross the globe. This is unprecedented data. The authors employed the well proven isotope balance formulation-the Craig Gordon evaporation model- to compute the evaporative water loss of lakes with respect to their inflows- i.e. E/I ratio. They authors then compare lakes accross the various geo/climatic regions based on isotope behaviors to identify key environmental factos that affect/control the isotope composition of the lakes. The manuscript merits publication in Nature Communications.

We would like to thank Reviewer 3 for the overall positive opinion about our work and for their time spent on the paper reading and reviewing. We answered all comments and provided the detailed explanations below.

Comment: However, there needs to be clarifications regards a few questions raised in the annotated PDF manuscript. The most important questions to be addressed are the following

Comment 27: 1. The computation that globally the E/I is equal to 0.2 looks a significant underestimation of evaporative water loss in catchments. This finding must be clarified. If this value represent the evaporative water loss with respect to inflows in lakes the value may be agreeable. However, this ratio underestimates the role of evaporative water loss in catchments if whole catchment is considered. The authors must clarify what the 0.2 fraction stands for.

Reply 27: We agree with Reviewer that it was not clearly described in the first version and made the correction in the abstract and main text. E/I is obtained from the isotope mass-balance determination and represents the evaporation losses of water inflows in the lake. This isotopic approach has been used to estimate fluxes regulating the water balance of lakes, including evaporation (E) to inflow (I) ratios (E/I) (*Gonfiantini 1986, Gibson et al. 1993, 2002, 2020; Kebede et al. 2009*).

L. 38-40: "Isotope mass-balance modelling showed that ca. 20 % of water inflow in global lakes is lost through evaporation and ca. 10 % of lakes in arid and temperate zones have extreme evaporative losses >40 % of the total inflow."

L. 79-80: "… by precipitation amount weighted inputs and evaporation losses of the lake inflows which…".

Comment 28: 2. ON the data selection approaches: The authors argue that datasets which show discrepancy in E/I computation when O and H were used have been eliminated from the dataset. However, as widely published and also urged by the authors (line 136-140) there discrepancy between O and H method a well-known problem. What percent of the dataset has been eliminated/not considered in the analysis must be reflected in the manuscript.

Reply 28: Studies showed that an agreement between δ^2H and $\delta^{18}O$-based calculations reflects the goodness of fit of the lake water balance to the overall mass-balance scenario (*Gibson and Reid 2014, Stadnyk and Holmes 2020*). Comparison between estimates based on two isotopes was used as a guideline to judge the uncertainty of the model. In general, estimates based on both isotope tracers agree within 10-20%. Previous studies have provided a detailed overview of potential errors associated with application of isotope-based models (*Gibson et al. 1993, Vystavna et al. 2018, Stadnyk and Holmes 2020*). Although absolute errors are difficult to evaluate, the estimates are likely comparable to standard physically based water balances, which has also been confirmed in other studies (*Gibson et al. 2002*). Overall, accuracy of annual water balance estimates for the current application is likely not better than 20±30%, although it is expected to be half an order of magnitude more precise for dual isotope comparative applications as was done in our study. The principal advantage of the current approach is that accuracy is significantly improved by using two tracers. The discrepancy between estimates obtained by two tracers can be due to:

1. $\delta^{18}O$ has a historically better precision quality than δ^2H because analysis of the deuterium was done using the indirect method before 2010s.
2. Isotope mass balance models generally assume the steady state conditions in headwater catchment and uncertainty is increasing in the case of non-steady state conditions and chain lakes or other more complex hydrological systems that can change the $^{18}O/^2H$ relationship and increase the discrepancy between $\delta^{18}O$- and δ^2H – based calculations (*Gibson and Reid 2014*).

In total ca. 50% lakes presented sufficient results of E/I calculated by both tracers. 35% of lakes had negative and 8% of lakes had E/I is > 1 indicating that the lakes were unbalanced, and the simplified conditions (steady-state model) cannot be applied. It needs the additional data or parameters adjustment that was out of the scope of the present study. 7% of lakes had only one tracer measured, or the results obtained between two tracers diverged. The latter was mainly lakes sampled before 1990. The full database will be available after the paper acceptance.

Comment 29: 3. ON comparing the 18O-2H at the Intersection of LEL and GMWL with the mean 18O-2H of Local Precipitation: The general finding that there is a difference between the two

is a significant finding! However for at least one case the reasoning behind such departure needs to be elaborated further or explained. The case is when the intersection point shows enrichment compared to the precipitation. The authors argue that is because of pre-evaporation within the catchment that makes intersection points to show higher values. However, as shown in Gonfiantini 1986 and Kebede et al., 2009; pre-evaporation of the inflowing water to the lakes would not alter the slope of the LEL. Evaporation within lakes only pushes the isotopic composition along the LEL without altering the slope. Therefore, the authors must find alternative explanation for the discrepancy between the two values.

Reply 29: Precipitation and other waters that have not undergone evaporation (such as most groundwaters) generally fall along a local meteoric water line (LMWL) having a slope of about 8, usually lying close to global meteoric water line (GMWL). Meteoric waters that undergone evaporation display systematic enrichment in both $\delta^{18}O$- and $\delta^{2}H$ resulting in divergence from the LMWL along evaporation lines (LEL) having slopes of less than 8 (often ranging from 4 to 6). Clustering of surface water in the catchment along a single well-defined LEL is applied for the headwater catchment *(Gibson et al. 1993*) and can be different for the complex hydrologically connected reservoirs. Displacement along the LEL from the point of the intersection of LEL and LMWL (or GMWL) varies within and between lakes in response to varying water balance (*Gibson et al. 1993*).

Comment 30: 3. On comparing isotope based E/I with environmental variables: The manuscript depicts the various environmental and climatic factors as controls on the isotopic composition. However, in my view some of these parameters are causally related to the E/I and others are just correlational. See the attached annotated PDF for details.

Annotated Comment from pdf: L. 167 I dont think control is the right term here. The authors investigated the co -relation between the isotopic compositions and the various environmental variables. The correlation doesn't necessary reflect the causal/mechanistic relations between isotope contents and the various variables. I suggest the authors clarify the above by mentioning this assessment is correlation.

Reply 30: Following Reviewer advice, the subsection title was revised to: **Climate and catchment-scale processes effect lake evaporation status.**

Our data showed that the relationship between isotope values (evaporative enrichment) and the studied variables is highly distinct (See Figure enclosed) and often polynomial depending on the climate type. Therefore, we have used random forest that is used in modelling predictions and behaviour analysis and is built on decision trees (*Breiman 2001*).

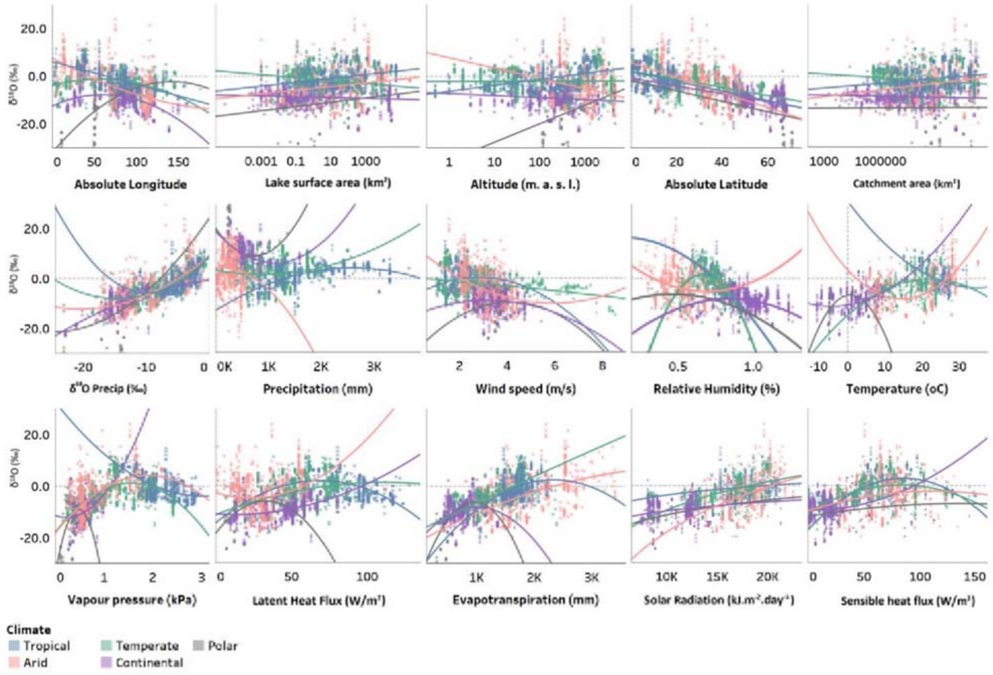

Figure. The scattered plots show the relation between oxygen-18, climate and catchment variables and its polynomial trends.

The random forest technique considers the instances individually, taking the one with most votes as the selected prediction (*Breiman 2001, Bouron et al. 2013, Buolesteix et al. 2014*). Each tree in the classifications takes input from samples in the initial dataset making it robust against autocorrelation, that is the problem for many other statistical approaches (*Breiman 2001, Boulesteix et al. 2014, Chen et al. 2017, Feng et al. 2017*). Features are then randomly selected and used in growing the tree at each node. Every tree in the forest should not be pruned until the end of the exercise when the prediction is reached decisively. In such a way, the random forest enables any classifiers with weak correlations to create a strong classifier (*Breiman 2001, Boulesteix et al. 2014*). The random sampling technique used in selecting the optimal splitting feature lowers the correlation and hence, the variance of the regression trees (*Breiman 2001, Boulesteix et al. 2014*). It improves the predictive capability of distinct trees in the forest. The sampling using bootstrap also increases independence among individual trees. Regression analysis is statistical process for estimating the relationships among variables and random forest is machine learning method capable of performing both regression and classification tasks (*Breiman 2001, Boulesteix et al. 2014*). Random Forest method can discover more complex relationships than regression analysis which is based on the correlation (*Breiman 2001, Boulesteix et al. 2014*) and became highly popular in many hydrological studies (i.e., *Baudron et al. 2013, Feng et al. 2017, Lu et al. 2018*) and described in the provided Supplementary information (**p. 9-14**).

Comment 31: For example, when the authors argue that temperature is not an important factor for polar lakes there is need for caution. This is because in the Criag-Gordon model used to compute E/I, temperature is implied in the equation.

Reply 31: We have used normalized isotope value ‘evaporative enrichment’ in lakes and not E/I for the model. Also, we excluded polar lakes and explained the reason in the text:

L. 193-199: “Selected catchment and climate variables in the (few) polar lakes had generally low explanatory power for $\Delta_{L\text{-}P}\ \delta^{18}O$ (R^2 = 4 %) since the isotopic composition of these

lakes are mainly indicative of water inputs than evaporation. Lake recharge in polar regions originates largely from melting of the cryosphere (snow, glacier, ice, soil permafrost) in high latitudes[25], polar lakes have high albedo from snow and ice cover which reduces absorption of solar energy and the exchange of water between the watershed and atmosphere[2]."

Comments from pdf file:

Comment 32: Page 2, L.39: This may be a significance underestimation. Typically 65% of the water is lost to evapotranspiration? If this finding is correct, then this is a significant new result. The authors need to elaborate how their finding agrees or disagrees with existing literature on the scale of evaporative water loss from catchments.

Reply 32: Explanation and correction are described in the comment above (Comment 27).

Comment 33: L. 45 – remove 'adverse'.

Reply 33: removed.

Comment 34: L. 49: do you mean storage?

Reply 34: corrected on storage.

Comment 35: L. 66 (word 'casual'): I wonder if the parameters to be discussed are causal or just corelations. Furthermore, comparing the isotope based (implicitly, mass transfer based evaporation estimation) with other independent parameters that for example the energy balance based evaporation estimation approaches (Bowen ratio, Penman, Presley Taylor, etc) would incorporate is commensurate to comparing mass transfer method with energy transfer methods.

Reply 35: Explanation and correction are in the answer on the general comment above (Comment 30). The word 'causal' was deleted as it can lead to misinterpretation.

Comment 36: L. 67-68: As isotope balance approaches is based on the physics of mass transfer derived by humidity gradient, wind speed is implicitly incorporated in the Crsig Gordon Turbulent parameters. Solar radiation was relevant if the isotope mass balance model rely on the energy balance approach.

Reply 36: Yes, we agree with the Reviewer that these parameters are included in the empirical models of evaporation and other hydroclimate processes estimation. We used these parameters as independent variables to explain the isotope value (evaporative enrichment) (details are in the method section of the Supplementary information) in lakes by the random forest machine learning tool (details are in the reply on Comment 30).

Comment 37: L. 76-79. ...this sentence is incomplete or not clear. Isotopic composition of Earth's lakes are contrled by preciptation amount wt input is correct, what about the role of evaporative water loss? Please rephrase this statement.

Reply 37: We agree with the reviewer and rephrase the sentence as following:

L. 76-79: ***"Our hypothesis was that the stable isotope compositions of Earth's lakes are controlled by precipitation amount weighted inputs and evaporation losses of the lake inflows which are controlled by distinctive combinations of regional climatic and catchment-scale environmental factors."***

Comment 38: L. 77: 'which are' instead of 'are'

Reply 38: corrected as proposed by the reviewer.

Comment 39: L.103 but the authors own finding shows m is greater than 5 as also argued by other authors (e.g. Kebede et al., 2009).

Reply 39: We agree with the reviewer and added the proposed reference *on Kebede et al. (2009).*

Comment 40: L.110-112 This is argument is not convincing. As shown in Gonfiantini, 1986 and various subsequent works the slope of the local evaporation line (LEL) would not be affected by the pre-enrichment of the isotopic composition of the waters in the catchment (See Kebede et al., 2009: Journal of Hydrology). Pre-enrichment would displace the composition of the catchment water along the LEL and subsequent evaporation of the same water in the lakes would displace the waters further along the LEL without altering the slope of the LEL and thereby without affecting the intersection between GMWL and LEL. The condition under which the slope of the local evaporation line and thereby the intersection between the LEL and GMWL depends on environmental conditions is, when there is no equilibrium between the isotopic composition of ambient vapor and the waters feeding the lakes, see figure 4 in Kebede et al., 2009. In my view the departure of the intersection point from the mean composition of the meteoric water depends on humidity conditions.

Reply 40: We thank the reviewer for his comment but our statement in Lines 110-112 refers to the intersection of the LEL with the GMWL and not to the slope. The text discusses the difference in the intersection $\delta^{18}O$ values per climate type highlighting that more positive $\delta^{18}O$ values were observed in the continental, temperate and tropical climates compared to all other types. The more positive values were not attributed to pre-enrichment due to evaporation but to the water storage in the lake catchments. We did not state that a change in the slope of the LEL because of this difference is expected which agrees with the reviewer's comment.

Comment 41: L.118-119 Same as above. Pre-evaporation within the catchment will not alter the slope of the LEL and thereby the foci of the intersection point.

Reply 41: detailed answer is provided in the previous comment (Comment 40).

Comment 42: L. 135: the isotopic composition of the vapor layer?

Reply 42: corrected.

Comment 43: L. 139-140 This argument/observation is not new. The authors can make reference to other existing evidence from regional or local studies.

Reply 43: These references were added:

Bowen, G.J., Putman, A., Brooks, J.R. et al. Inferring the source of evaporated waters using stable H and O isotopes. Oecologia 187, 1025–1039 (2018).

Vallet-Colomb, C., Gasse, F., Sonzogni, C. Seasonal evolution of the isotopic composition of atmospheric water vapour above a tropical lake: deuterium excess and implication for water recycling. Geochim. Cosmochim. Acta 72, 661-674 (2008).

Comment 44: L. 151 (refer to 'albedo') what about the effect of seasonality forwarded by Gibson et al works

Reply 44: details provided in the reply on Comment 12.

Comment 45: L. 165: E/I of 0.2 indicates 20% of the inflow water is lost to evaporation in the lake. How this translates to the lake catchment is not convincing/clear. I thus suggest keeping

the evaporative loss projections to the lakes not to their catchments. The finding also contradicts other findings that argue that catchment evapotranspiration is the dominant pathway of water loss.

Reply 45: Vapour loss mechanisms, including canopy interception, evaporation from soil, evaporation from wet surfaces, and transpiration by plants, are often referred to collectively as evapotranspiration (ET) (*Li et al. 2019*). However, in our study, we didn't target the separation of ET on evaporation and transpiration as it was already done by *Jasechko et al (2013)* and *Gibson et al. (2021)* who demonstrated that transpiration fluxes are dominating with the higher range depending on the region (30-90%). Recent study of *Gibson et al. (2021)* in Canada showed on 162 stations located in the different climate regions that the E/I in the catchment is ca. 20% that is in the agreement with *Jasechko et al. (2013)* and our calculations. Evaporation-dominated water loss in watersheds is commonly associated with open water, lichen and moss, and wetlands that is mainly characteristic for continental climate and explains the lower LEL slope in these catchments. Note that lichen and mosses that are typical for the wetland area are non-vascular, and therefore respiration is accomplished by evaporation rather than transpiration *(Price et al. 2009)*.

Comment 46: L. 168 – to remove 'isotope' word

Reply 46: corrected.

Comment 47: L. 222-223 same as above, the 20% loss is for the evaporative water loss with respect to inflow into lakes. It doesn't represent the evaporative water loss in catchments.

Reply 47: We agree and changed the sentence to be clear for the reader.

L.223: "At the global scale, evaporation losses accounted for ca. 20 % of the water inflows into the lake".

Comment 48: L. 240-242 This argument sounds tricky, Basically, there are three evaporation estimation methods- mass transfer based whereby humidity, wind-speed and temperature plays role; energy balance whereby the balance of incoming and outgoing radiation is used and the combined approach (e.g Penman method) which combines the two. The Criag-Gordon model is basically based on the mass transfer physics. So comparing Craig-Gordon based E/I with the other conventional is trivial. In fact the Penman Monteith evaporation model for open waters accounts for mass transfer which is the basis of the isotope balance method. So this argument must be clarified.

Reply 48: We agree with the reviewer. However, our objective is not to compare the isotope balance method with other conventional evaporation methods but to demonstrate that other variables incorporated in these models play an important role in the evaporation water losses with respect to inflow. The sentence was re-written as follows:

L 240-243: *"We showed that not only are relative humidity and air temperature indicative for predicting the isotopic composition of global lakes, but also variables related to surface energy processes, that are incorporated in other conventional evaporation models (e.g. Penman)".*

Comment 49: L. 285 – 287. The criteria of selecting the E/I values obtained from the comparison of balances computed based on H and O is tricky. In fact as argued by the authors (line 136-140) in many cases there is discrepancy between the computations base H and O are different (in other words. the theoretical slope departs from the computed slope). The authors must specify

what proportion of the data has been eliminated from being considered in the E/I estimation and the subsequent analysis.

Reply 49: the reply is provided earlier in Comment 29.

REFERENCES:

Baudron, P. et al. Identifying the origin of groundwater samples in a multi-layer aquifer system with random forest classification. *J. Hydrol.* **499**, 303–315 (2013).

Boulesteix, A.L. & Schmid, M. Machine learning versus statistical modeling. *Biom. J.* **56**, 588–593 (2014).

Breiman, L. Random forests. *Mach. Learn.* **45**, 5–32 (2001).

Chen, G. Long, T. Xiong, J. & Bai, Y. Multiple random forests modelling for urban water consumption

forecasting. *Water Resour. Manag.* **31**, 4715–4729 (2017).

Craig, H. & Gordon, L. I. Deuterium and oxygen 18 variations in the ocean and the marine atmosphere. (1965).

Duan, Z., & Bastiaanssen, W. G. M. A new empirical procedure for estimating intra-annual heat storage changes in lakes and reservoirs: review and analysis of 22 lakes. *Remote Sens. Environ.* **156**, 143-156 (2015).

Evaristo, J, & McDonnell, JJ. A role for meta-analysis in hydrology. *Hydrol. Process.* 1–4 (2017)

Feng, Y. Cui, N. Gong, D. Zhang, Q. & Zhao, L. Evaluation of random forests and generalized regression neural networks for daily reference evapotranspiration modelling. *Agric. Water Manag.* **193**, 163–173 (2017).

Gibson, J.J., Edwards, T.W.D., Bursey, G.G. & Prowse, T.D. Estimating evaporation using stable isotopes: quantitative results and sensitivity analysis for two catchments in northern Canada. *Nord. Hydrol*. **24**, 79-94 (1993).

Gibson, J.J., Prepas, E.E. & McEachern, P. Quantitative comparison of lake throughflow, residency, and catchment runoff using stable isotopes: modelling and results from a survey of boreal lakes. *J. Hydrol.* **262** (2002).

Gibson, J.J., & Reid, R. Water balance along a chain of tundra lakes: A 20-year isotopic perspective. *J. Hydrol.* **519**, 2148-2164 (2014).

Gibson, J.J., et al. Isotopic constraints on water balance and evapotranspiration partitioning in gauged watersheds across Canada. *J. Hydrol. Reg. Stud.* **37**, 100878 (2021).

Gonfiantini, R. Environmental isotopes in lake studies P. Fritz, J.Ch. Fontes (Eds.), Handbook of Environmental Isotope Geochemistry, vol. 13, Elsevier, New York (1986), pp. 113-168

Jasechko, S., et al. Terrestrial water fluxes dominated by transpiration. *Nature Commun.* **496**, 347–350 (2013).

Jasechko, S., Gibson, J.J. & Edwards, T.W.D. Stable isotope mass balance of the North American Great Lakes. *J. Great Lakes Res.* **40**, 336-346 (2014).

Jasechko, S. Global isotope hydrogeology—review. *Rev. Geophys*. **835-965**, 57 (2019).

Isokangas, E., Rozanski, K., Rossi, P.M., Ronkanen, A.-K. & Klove B. Quantifying groundwater dependence of a sub-polar lake cluster in Finland using an isotope mass balance approach. *Hydrol. Earth Sys. Sci.* **19**, 1247-1262 (2015).

Kebede, S., Travi, Y. & Rozanski, K. The δ18O and δ2H enrichment of Ethiopian lakes. *J. Hydrol.* **365**, 173-182 (2009).

Kourzeneva, E., Asensio, H., Martin, E., & Faroux, S. Global gridded dataset of lake coverage and lake depth for use in numerical weather prediction and climate modelling. *Tellus A* **64**, 15640 (2012).

Li X., et al. A simple and objective method to partition evapotranspiration into transpiration and evaporation at eddy-covariance sites. *Agric. Forest Meteorol.* 171-182 (2019).

Lu, X., et al. Daily pan evaporation modeling from local and cross-station data using three tree-based machine learning models. *J. Hydrol.* **566**, 668–684 (2018).

Messager, M.L., Lehner, B., Grill, G., Nedeva, I. & Schmitt, O. Estimating the volume and age of water stored in global lakes using a geo-statistical approach. *Nat. Commun.* **7**, 13603 (2016).

Pope, A. et al. Estimating supraglacial lake depth in West Greenland using Langsat 8 and comparison with other multispectral methods. *The Cryosphere* **10**, 15-27 (2016).

Price, J.S., Edwards, T.W.D., Yi, Y. & Whittington P.N. Physical and isotopic characterization of evaporation from Sphagnum moss. *J. Hydrol.* **369**, 175-182 (2009).

Pyrchla, K., Dworaczek, A. & Wiezbicka, A. A method estimating the depths of shallow water based on the measurements of upwelling irradiane. *J. Coastal Conserv*. **22**, 777-786 (2018).

Rozanski, K., Froehlich, K., Mook, W.G. Environmental isotopes in the hydrological cycle. Principles and Applications 39, Volume III. Surface water. Technical documents in hydrology. Edited by W.G. Mook. Published by UNESCO (2001).

Sivapalan, M., & Bloschl, G. Time scale interactions and the coevolution of humans and water. *Water Resour. Res*. **51**, 6988–7022 (2015).

Sneed, W.A. & Hamilton, G.S. Validation of a method for determining the depth of glacial melt ponds using satellite imagery. *Ann. Glaciol.* **52,** 15-22 (2011).

Stadnyk, T. A., & Holmes, T. On the value of isotope-enabled hydrologic model calibration. *Hydrol. Sci. J.* **65(9)**, 1525–1538 (2020).

Zhao, G., Gao, H., & Cai, X. (2020). Estimating lake temperature profile and evaporation losses by leveraging MODIS LST data. *Remote Sens. Environ.* **251**, 112104.

Vallet-Colomb, C., Gasse, F., Sonzogni, C. Seasonal evolution of the isotopic composition of atmospheric water vapour above a tropical lake: deuterium excess and implication for water recycling. Geochim. Cosmochim. Acta 72, 661-674 (2008).

Vystavna, Y., et al. Isotopic response of run-off to forest disturbance in small mountain catchments. *Hydrol. Process.* **32** (24), 3650-3661 (2018).

Reviewers' Comments:

Reviewer #1:
Remarks to the Author:
The authors have well addressed all my comments and revised the manuscript accordingly. I think the manuscript is much improved, so I do not have any more comments to add. Good luck.

Reviewer #2:
Remarks to the Author:
The authors addressed my and the other reviewers' comments thoroughly and in much detail. The revised version can be published in current form. I have no further comments.

Reviewer #3:
Remarks to the Author:
The authors have satisfactorily addressed all the issues I raised.

I recommend the publication of the manuscript.

REVIEWER COMMENTS

We would like to acknowledge the reviewers for the positive opinion and support of our work, but also comments and ideas that helped to improve the manuscript.

Dr Yuliya Vystavna on behalf of all authors

REVIEWERS' COMMENTS

Reviewer #1 (Remarks to the Author):

The authors have well addressed all my comments and revised the manuscript accordingly. I think the manuscript is much improved, so I do not have any more comments to add. Good luck.

Reply: Thank you very much for your support and important points raised during the reviewing of the paper.

Reviewer #2 (Remarks to the Author):

The authors addressed my and the other reviewers' comments thoroughly and in much detail. The revised version can be published in current form. I have no further comments.

Reply: Thank you very much for the time spent on our study and ideas that improved the paper.

Reviewer #3 (Remarks to the Author):

The authors have satisfactorily addressed all the issues I raised.

I recommend the publication of the manuscript.

Reply: Thank you very much for the attention to our study, comments and ideas that improved the paper.
